# An exploration into the causal relationships between educational attainment, intelligence, and wellbeing: an observational and two-sample Mendelian randomisation study

**DOI:** 10.1038/s44184-024-00066-x

**Published:** 2024-05-09

**Authors:** J. M. Armitage, R. E. Wootton, O. S. P. Davis, C. M. A. Haworth

**Affiliations:** 1 Wolfson Centre for Young People’s Mental Health, Cardiff University, Cardiff, Wales UK; 2 School of Psychological Science, University of Bristol, Bristol, UK; 3 Nic Waals Institute, Lovisenberg Diaconal Hospital, Oslo, Norway; 4 Bristol Medical School (PHS), University of Bristol, Bristol, UK

**Keywords:** Epidemiology, Genetics research, Psychology

## Abstract

Educational attainment is associated with a range of positive outcomes, yet its impact on wellbeing is unclear, and complicated by high correlations with intelligence. We use genetic and observational data to investigate for the first time, whether educational attainment and intelligence are causally and independently related to wellbeing. Results from our multivariable Mendelian randomisation demonstrated a positive causal impact of a genetic predisposition to higher educational attainment on wellbeing that remained after accounting for intelligence, and a negative impact of intelligence that was independent of educational attainment. Observational analyses suggested that these associations may be subject to sex differences, with benefits to wellbeing greater for females who attend higher education compared to males. For intelligence, males scoring more highly on measures related to happiness were those with lower intelligence. Our findings demonstrate a unique benefit for wellbeing of staying in school, over and above improving cognitive abilities, with benefits likely to be greater for females compared to males.

## Introduction

In most societies, education provides young people with the knowledge, skills, and socialisation necessary to prepare for adult life. The number of years spent in schooling can therefore be an important determinant of later outcomes and functioning, as evidenced by greater occupational status and income, marriage, and health^1,2^. The extent to which some of these relationships are causal, however, remains less clear. Educational attainment has been shown to causally impact smoking, sedentary behaviours, and Body Mass Index^3^, as well as the risk of suicide attempts, insomnia, and major depressive disorder^4^. Yet also fundamental to health and success is wellbeing^5^, but the causal impact of educational attainment on wellbeing remains unexplored.

Wellbeing is broadly defined as relating to feelings of satisfaction and happiness^6^. Observational studies investigating the impact of educational attainment on wellbeing have produced mixed results, with evidence to suggest both direct^7^ and indirect effects^2^, as well as both positive and negative influences^8^. Indirect effects of education refer to those that occur via mechanisms other than education itself, such as through income, employment, marriage, children, or health^2^. Positive indirect influences of educational attainment on wellbeing have been noted largely through income, with males and females experiencing benefits of education through increased earnings^2^. Some sex differences have been noted for other indirect paths, like employment, whereby the wellbeing of educated males but not females is heightened through being employed^2^. When these indirect paths are not accounted for, associations between educational attainment and wellbeing have been shown to be negative^2^, suggesting that education exerts its benefits through many different channels. Most of the findings to date however, are based on samples from Australia, with just one study to date investigating associations in a UK sample^9^. This study found little effect of educational attainment on happiness, and little impact of a reform that raised the school leaving age. No study has jointly considered the role of intelligence and schooling on overall wellbeing.

Intelligence is often used to refer to the many facets of cognitive functioning, including memory and learning, processing speed, as well as abstract, verbal, and spatial reasoning^10^. These abilities are all interrelated and highly correlated with educational attainment^11^, yet observational findings have suggested associations with wellbeing may differ to those found for educational attainment. In particular, associations between intelligence and wellbeing are often positive, but switch to negative after accounting for other correlated factors like income and parental education^12,13^. This has been suggested to reflect the greater expectations that come with being highly intelligent and a higher earner^12^. The correlational nature of these studies, however, does not permit causal inferences for either the direct or indirect effects.

Determining whether associations are causal or driven by unobserved or imprecisely measured confounders is crucial to establishing true and unbiased effects. Mendelian randomisation (MR) is a study design that uses summary-level genetic data to assess potentially causal relationships^14^. The methods of MR enable control over both confounding and reverse causality, and can be extended to multivariable MR when teasing apart the combined and independent effects of highly correlated variables, like educational attainment and intelligence.

So far, MR studies have revealed that despite their high correlation, intelligence and educational attainment exert independent causal effects on some health and economic outcomes^3,11^. Extending such findings to wellbeing could therefore help to inform best practice for maximising optimal functioning. In particular, if associations between educational attainment and wellbeing are largely accounted for by intelligence, policy makers would benefit from focusing less on keeping students in higher education, and more on improving cognitive abilities. If, however, educational attainment exerts a direct positive impact on wellbeing, policy makers would benefit from extending requirements to remain in further education.

Few studies to date have studied causal associations with wellbeing^15^, and even fewer have made use of the latest genetic instrument for wellbeing^16^. This instrument combines four wellbeing related traits (life satisfaction, positive affect, neuroticism, and depressive symptoms), which are referred to collectively as the wellbeing spectrum. This phenotype has been associated with more genetic signals than previous genetic analyses based on positive affect and life satisfaction alone^17^. The first part of this study therefore makes use of this instrument for wellbeing in univariable and multivariable MR to test for the first time, whether educational attainment and intelligence are causally and independently related to wellbeing. Bidirectional associations are also explored as findings have shown that wellbeing not only results from successful outcomes, but it also precedes them^18^. Understanding whether associations work both ways could therefore highlight important paths to improving overall functioning.

One drawback of using the MR design is that estimates are not time bound, meaning implications for intervention may be less clear. The second part of this study therefore supplements genetic findings using longitudinal observational data. The aim is to understand the impact of educational attainment and intelligence on wellbeing in emerging adulthood, a critical life stage for establishing identity and adult mental health. Such analyses aimed to also further scrutinise the relationship between educational attainment and wellbeing to clarify possible sex differences^8,19^, non-linear trends^20^, and moderating effects of intelligence.

## Results

### Univariable MR testing causal associations between educational attainment and intelligence

Prior to investigating effects on wellbeing, univariable MR was first used to confirm the bidirectional relationship between educational attainment and intelligence^11^. Analyses revealed strong causal effects of educational attainment on intelligence, and vice versa (Supplementary Table 1). Effect sizes were twofold greater for educational attainment on intelligence, aligning with previous findings^11^. There was also strong evidence of heterogeneity in the causal estimates for both directions, also replicating previous findings.

### Univariable MR testing causal associations between educational attainment and wellbeing

**Fig. 1:**
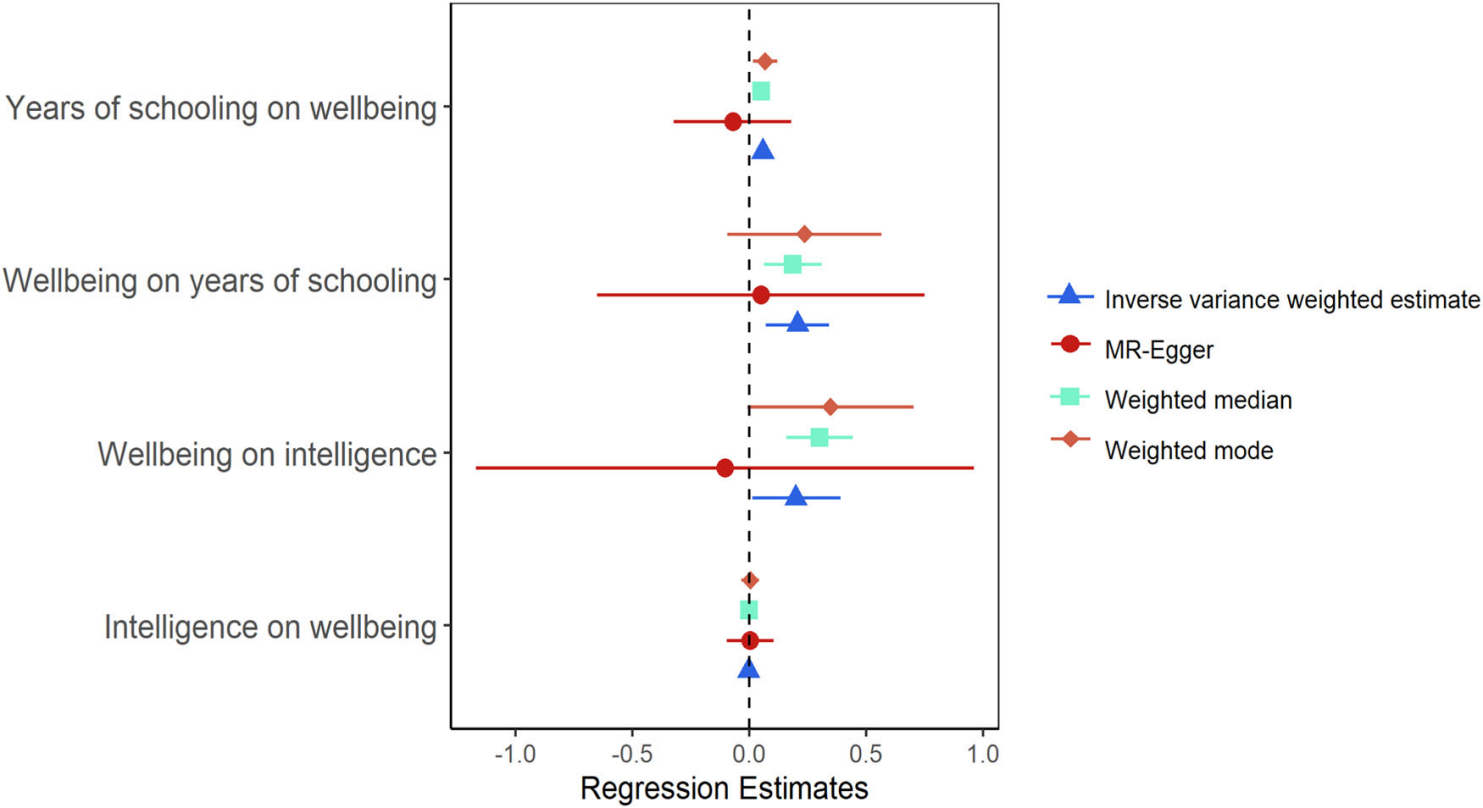
Comparison of the main and sensitivity univariable MR analyses. The main analysis is the inverse variance weighted estimate. The MR-Egger, Weighted median, and weighted mode represent sensitivity analyses.

Univariable MR analyses exploring total causal effects of educational attainment on wellbeing provided evidence of a small positive impact (Fig. 1). For every standard deviation (SD) increase in years of schooling, which equates to 3.6 years of schooling, there was a 0.057 (95% CI = 0.042, 0.074) increase in wellbeing, as assessed using the wellbeing spectrum. There was also evidence of a causal impact of wellbeing on educational attainment. Analyses revealed that a SD increase in wellbeing predicted a 0.206 (95% CI = 0.071, 0.341) increase in the number of years schooling (see Supplementary Table 2).

Neither of these findings replicated using MR-Egger (see Fig. 1), which as explained in the methods, is unlikely to be a result of directional pleiotropy as the MR-Egger intercept did not differ from zero. The funnel plots also provided evidence of balanced pleiotropy and there was no evidence in the forest plots to suggest that associations were strongly driven by one single-nucleotide polymorphism (SNP) (see Supplementary Figs. 1 and 2). Steiger filtering revealed that all educational attainment SNPs were more associated with educational attainment than wellbeing, and sensitivity analyses removing 4 wellbeing SNPs that explained more of the variance in educational attainment revealed consistent results (see Supplementary Table 3), suggesting minimal bias from reverse causation. Instead, given the large confidence intervals and the low regression dilution statistic (See Supplementary Table 4), it is likely that MR-Egger results can be accounted for by measurement error.

Analyses predicting subjective happiness and life satisfaction using the trait-specific estimates from the model-averaging GWAMA^16^ revealed largely consistent findings, with effect sizes most similar to the full wellbeing spectrum for analyses predicting life satisfaction (see Supplementary Tables 5 to 6). As anticipated, results for neuroticism and depression produced associations in the opposite direction (Supplementary Tables 7 and 8).

### Univariable MR testing causal associations between intelligence and wellbeing

Univariable MR analyses investigating associations between intelligence and wellbeing revealed no causal effects of intelligence on wellbeing, but evidence of a causal impact of wellbeing on intelligence (Fig. 1). Effect sizes were similar to those found for educational attainment, with a SD increase in wellbeing predictive of a 0.199 (95% CI = 0.014, 0.390) increase in intelligence (Supplementary Table 2). This did not replicate using MR-Egger, but was consistent across other sensitivity analyses. As per analyses on educational attainment, findings also provided no evidence of bias due to directional horizontal pleiotropy (see Supplementary Fig. 3). Analyses conducted after removing 11 wellbeing SNPs following Steiger filtering also revealed largely consistent results (see Supplementary Table 3).

### Multivariable MR

**Fig. 2:**
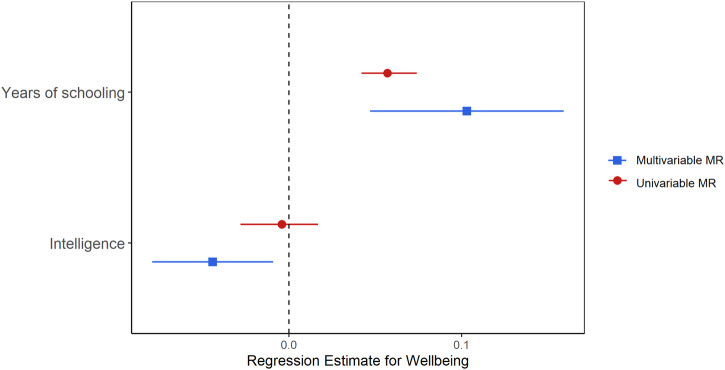
Comparison of univariable and multivariable MR analyses predicting wellbeing based on IVW MR estimates. This figure shows that years of schooling has positive independent (multivariable) and total (univariable) causal effects on wellbeing. In contrast, intelligence has negative independent (multivariable) but not total (univariable) causal effects on wellbeing.

Results from the multivariable MR analysis revealed independent causal effects of both educational attainment and intelligence on wellbeing (see Fig. 2), however, findings were in the opposite direction to one another. For educational attainment, a one SD increase in years of schooling (3.6 years) predicted a 0.103 (95% CI = 0.05, 0.16) increase in wellbeing, controlling for the effects of intelligence, while intelligence predicted a 0.04 (95% CI = −0.08, −0.01) decrease in wellbeing, controlling for years of schooling. These findings were both larger than those found in the univariable models and were generated despite relatively weak instruments (F-statistic = 7.94 for intelligence and F-statistic = 7.23 for educational attainment). These F-statistics are lower than those in the univariable analyses due to estimating the impact of the SNPs on one exposure, conditioning on the other^21^.

Findings from the multivariable MR-Egger analyses produced the same pattern of results as above for both exposures (Table 1), and the MR-Egger intercept provided no evidence of directional pleiotropy. All univariable and multivariable MR findings also remained after adjustment for multiple testing using the Benjamini–Hochberg procedure^22^. Raw p-values are therefore reported to ensure consistency with the wider MR literature^23^.

**Table 1:** Comparison of total and independent effects of educational attainment and intelligence on wellbeing

		Causal effect estimates		Heterogeneity statistics		
	*N* SNPs	β (95% CI)	*P*	*Q*	df	*P*
Years of schooling on wellbeing						
Total effects						
IVW	54	0.057 (0.042, 0.074)	5.18E-13	336.7	53	<0.001
MR-Egger	54	−0.071 (−0.323, 0.180)	5.81E-01	330.1	52	<0.001
MR-Egger intercept	54	0.002 (−0.002, 0.006)	0.313	-	-	-
Independent effects						
IVW	151	0.103 (0.047, 0.159)	4.69E-04	766.7	149	<0.001
MR-Egger	151	0.064 (−0.006, 0.135)	0.075	751.4	148	<0.001
MR-Egger intercept	151	0.001 (−0.010, 0.003)	0.082	-	-	-
Intelligence on wellbeing						
Total effects						
IVW	126	−0.004 (−0.028, 0.017)	0.713	688.5	125	<0.001
MR-Egger	126	0.003 (−0.096, 0.103)	0.946	688.4	124	<0.001
MR-Egger intercept	126	−0.001 (−0.003, 0.001)	0.883	-	-	-
Independent effects						
IVW	151	−0.044 (−0.079,−0.009)	0.014	766.7	149	<0.001
MR-Egger	151	−0.075 (−0.124, −0.026)	0.003	751.4	148	<0.001
MR-Egger intercept	151	0.001 (−0.010, 0.003)	0.082	-	-	-

Analyses conducted using the educational attainment discovery and replication cohort (*n* = 293,723). Intercept estimates for the effects of intelligence and years of schooling on wellbeing are the same within the multivariable MR model as there is only one intercept.

*IVW* Inverse variance weighted estimate, this assumes no pleiotropy.

To further test the bi-directional relationship between educational attainment and wellbeing, we performed an additional multivariable MR analysis in which we investigated intelligence and well-being on years of schooling. Findings revealed that wellbeing was independently associated with years of schooling after accounting for intelligence, predicting a 0.193 (95% CI = 0.07, 0.31) increase in years of schooling, and intelligence was independently associated with years of schooling after accounting for wellbeing, predicting a 0.44 (95% CI = 0.40, 0.48) increase in years of schooling.

### Observational findings

#### Descriptives

Observational analyses were conducted using the Avon Longitudinal Study of Parents and Children (ALSPAC^24^). Among participants with data on educational attainment, intelligence, and wellbeing, approximately 66.7% had a university degree. Individuals who had a university degree scored significantly higher on the intelligence test at 8 years old (mean = 112.21, SD = 14.75, range = 62−148) compared to individuals without a university degree (mean = 99.07, SD = 14.93, range = 45−138), according to a Welch two sample *t* test, t(1879) = −22.2, *p* < 0.001.

Subjective happiness scores in the samples averaged 4.89 (range = 1 to 7), while life satisfaction scores averaged 24.25 (range = 5 to 35). Happiness scores were not significantly different among those with (mean = 4.89, SD = 1.27) or without (mean = 4.89, SD = 1.31) a university degree, but those with a degree had significantly higher life satisfaction scores (mean = 24.78, SD = 6.65) compared to those without a degree (mean = 23.09, SD = 7.36), t(2591) = 6.99, *p* < 0.001. Further information to wellbeing, educational attainment, and intelligence can be found in the supplementary (see Supplementary Tables 9 and 10).

### Testing linear associations

**Fig. 3:**
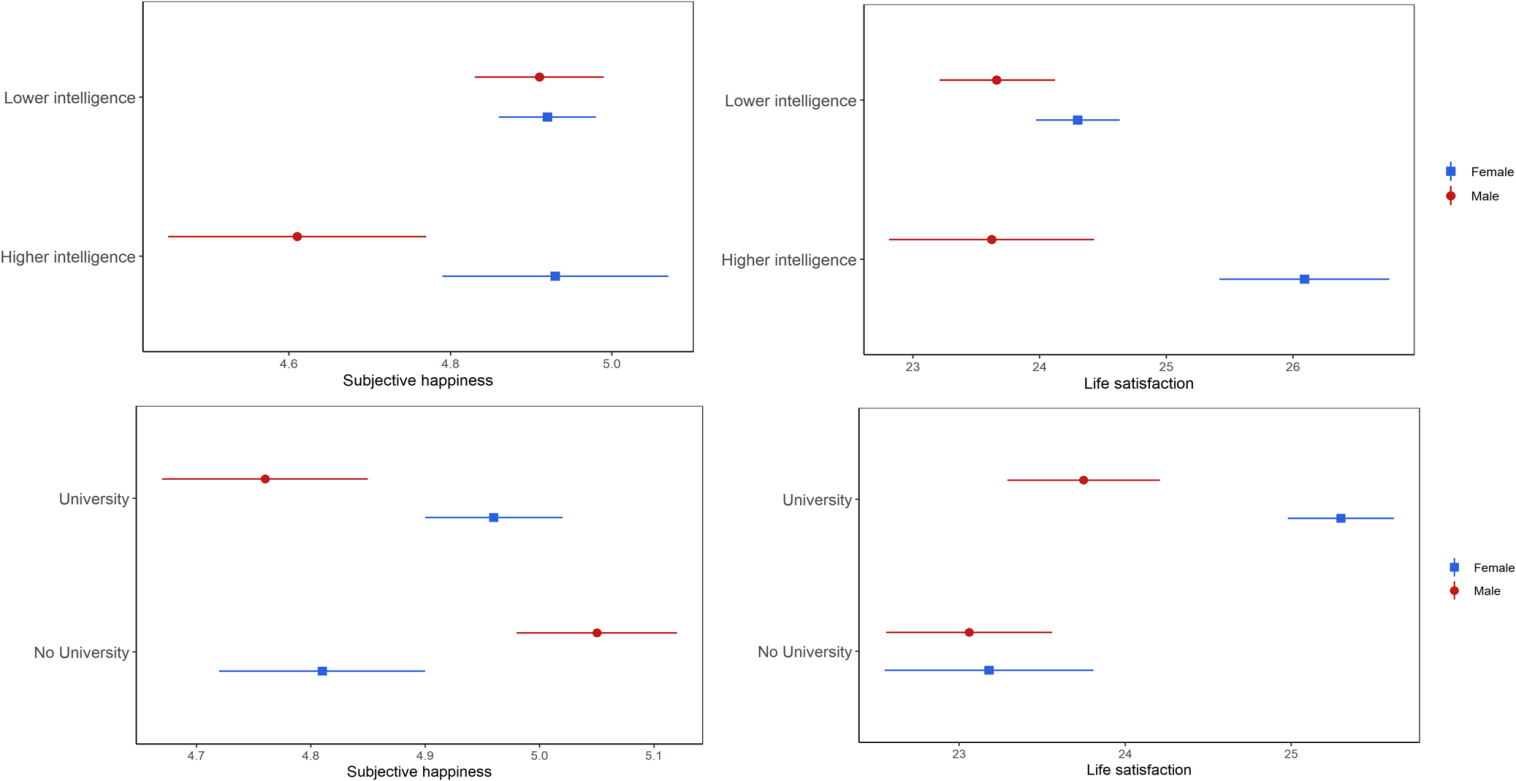
Interactive effects of sex on observational associations between educational attainment and wellbeing, and between intelligence and wellbeing. This figure shows differences between male and female subjective happiness and life satisfaction for those with higher intelligence (1 SD above the mean), and differences in subjective happiness for those with and without a university degree, and for life satisfaction for those with a degree.

Analyses revealed that higher educational attainment, indexed by having at least a university degree, was not associated with subjective happiness, but did predict increased life satisfaction (Table 2). After including adjustments for main and interactive effects of sex, findings showed that females who completed university had significantly higher life satisfaction than those who did not, with differences appearing greater than those noted between males with and without a degree (see Fig. 3). For subjective happiness, the direction of effects was the opposite for the two sexes, with females more likely to experience positive benefits to their subjective happiness if they completed university, whereas male graduates were more at risk of lower subjective happiness (see Fig. 3). These opposing results likely explain the absence of effects noted in models unadjusted for sex.

**Table 2:** Observational regression results assessing linear associations between educational attainment and wellbeing, and between intelligence and wellbeing

	Unadjusted		Adjusted using IPW		Adjusted using multiple imputation (*n* = 4298)		Complete cases (*n* = 2844)	
	β (95% CI)	*P*	β (95% CI)	*P*	β (95% CI)	*P*	β (95% CI)	*P*
Subjective happiness								
*Model 1*								
University degree	0.003 (−0.063, 0.070)	0.920	−0.020 (−0.102, 0.061)	0.624	−0.016 (−0.078, 0.046)	0.615	−0.022 (−0.098, 0.055)	0.581
*Model 2*								
Intelligence	−0.002 (−0.004, −0.000)	0.038	−0.003 (−0.005, −0.002)	4.58E-06^a^	−0.003 (−0.004, −0.000)	0.008^a^	−0.002 (−0.005, −0.000)	0.033
*Model 3*								
University degree	−0.226 (−0.339, −0.113)	9.18E-05^a^	−0.218 (−0.352, −0.084)	0.001^a^	−0.242 (−0.350, −0.136)	8.54E-06^a^	−0.219 (−0.347, −0.091)	7.98E-04^a^
Sex	−0.186 (−0.298, −0.075)	0.001^a^	−0.095 (−0.213, 0.022)	0.111	−0.184 (−0.289, −0.078)	6.63E-04^a^	−0.139 (−0.270, −0.009)	0.036
University degree*Sex	0.347 (0.208, 0.486)	1.05E-06^a^	0.308 (0.139, 0.477)	3.60E-04^a^	0.344 (0.212, 0.475)	3.08E-07^a^	0.307 (0.147, 0.466)	1.69E-04^a^
*Model 4*								
Intelligence	−0.005 (−0.009, −0.002)	0.003^a^	−0.003 (−0.005, −0.001)	0.003^a^	−0.006 (−0.009, −0.002)	4.27E-04^a^	−0.004 (−0.008, −0.000)	0.027
Sex	−0.475 (−0.957, 0.006)	0.053	−0.136 (−0.159, 0.423)	0.364	−0.493 (−0.913, −0.072)	0.022	−0.253 (0.760, 0.254)	0.329
Intelligence*Sex	0.005 (0.000, 0.001)	0.029	0.000 (−0.003, 0.002)	0.670	0.005 (0.001, 0.009)	0.013^a^	0.003 (−0.002, 0.008)	0.223
Life satisfaction								
*Model 5*								
University degree	0.241 (0.176, 0.307)	7.44E-13^a^	0.200 (0.118, 0.281)	1.66E-06^a^	0.231 (0.169, 0.294)	3.00E-15^a^	0.223 (0.147, 0.299)	1.04E-08^a^
*Model 6*								
Intelligence	0.005 (0.002, 0.007)	3.66E-05^a^	0.004 (0.003, 0.006)	2.66E-08^a^	0.004 (0.002, 0.006)	5.27E-06^a^	0.004 (0.002, 0.006)	2.40E-04^a^
*Model 7*								
University degree	0.076 (−0.036, 0.188)	0.185	0.015 (−0.119, 0.148)	0.826	0.091 (−0.015, 0.197)	0.092	0.070 (−0.056, 0.197)	0.276
Sex	−0.021 (−0.131, 0.089)	0.701	−0.034 (−0.151, 0.083)	0.568	−0.008 (−0.113, 0.097)	0.883	−0.008 (0.137, 0.121)	0.903
University degree*Sex	0.249 (0.111, 0.388)	4.07E-04^a^	0.281 (0.112, 0.449)	0.001^a^	0.214 (0.083, 0.345)	0.001^a^	0.235 (0.077, 0.393)	0.004^a^
*Model 8*								
Intelligence	0.002 (−0.002, 0.005)	0.340	0.004 (0.002, 0.006)	4.21E-05^a^	0.002 (−0.001, 0.005)	0.206	0.002 (−0.001, 0.006)	0.174
Sex	−0.418 (−0.898, 0.061)	0.087	0.182 (−0.115, 0.480)	0.230	−0.304 (−0.723, 0.115)	0.155	−0.199 (−0.703, 0.304)	0.439
Intelligence*Sex	0.005 (0.000, 0.010)	0.019^a^	−0.001 (−0.003, 0.002)	0.923	0.004 (0.000, 0.008)	0.035	0.003 (−0.001, 0.008)	0.158

^a^FDR. IPW= Inverse probability weighting. Sex coded as 0=Male and 1=Female, analyses therefore used male as the reference. In unadjusted models and models adjusted for IPW, *n* = 3788 for educational attainment and *n* = 3179 for intelligence.

Unadjusted models exploring the impact of intelligence revealed that as intelligence scores increased, subjective happiness declined, while life satisfaction increased (Table 2). After adding an interaction term between intelligence and sex, associations with life satisfaction remained, and associations with subjective happiness became positive. This suggests a moderating effect of sex, which is supported by plots of the findings (see Fig. 3). Males scoring more highly on the subjective happiness scale were those with lower intelligence scores (see Fig. 3).

All findings, including those for intelligence and educational attainment, remained after adjustment for multiple testing, and all findings replicated after adjustment for attrition (see Table 2 for adjusted results, and Supplementary Table 11 for unstandardised estimates).

### Testing non-linear associations and moderating effects

When testing the relationship between intelligence and wellbeing for non-linearity, there was no evidence to suggest that non-linear models fit the data better than the linear models (see Supplementary Table 12). There was also no clear evidence to suggest moderating effects of educational attainment, with no strong interactions found between educational attainment and intelligence in analyses predicting subjective happiness (β = 0.001, SE = 0.003, *p* = 0.721) or life satisfaction (β = 0.024, SE = 0.018, *p* = 0.197). Analyses also revealed that family income is unlikely to explain associations between educational attainment and wellbeing, and between intelligence and wellbeing (see Supplementary Table 13).

## Discussion

This study was the first to combine genetic and observational data to test for causal associations between educational attainment, intelligence, and wellbeing. The MR results suggest that the relationship between educational attainment and wellbeing is bidirectional, with the magnitude of effects greater for wellbeing on educational attainment than vice versa. Findings also revealed that the causal and protective effect of staying in school is independent of intelligence, but may be greater for females relative to males.

Investigations into intelligence showed that wellbeing has a positive causal impact on intelligence, but intelligence a negative impact on wellbeing. These negative effects were only found after adjusting for educational attainment, implying either a direct and independent role, or that independent effects are in the opposite direction to the combined effects. Observational findings confirmed the direction of this effect for associations with subjective happiness but not life satisfaction, however, as per educational attainment, there were underlying sex differences. Together the findings stress the importance of staying in education over and above cognitive abilities for wellbeing.

Our MR finding that individuals who are genetically inclined to stay on and complete more years of schooling will have greater wellbeing was implied in previous observational studies^1,19^ but not in a previous MR study^9^. The previous MR study found little impact of educational attainment on happiness. This was not found using positive affect in our MR analyses, however, previous findings do align with the current observational findings. These suggested that completing more years of schooling may positively impact life satisfaction but not happiness, however, males and females may be affected differently. The previous MR study by Davies and colleagues^9^ adjusted for sex differences, which likely explains the different results to our MR findings.

Similar sex differences to our study have been reported previously in observational studies, with associations between schooling and happiness stronger among females relative to males^8^. This study in combination with the present findings suggest that females gain more to their wellbeing from continuing their education compared to males. One explanation for this could be due to underlying differences in socialisation.

Studies have shown that socialising has a greater impact on happiness among females relative to males^25^. Education has been referred to as an “institutionalised form of social resource”^4^ and is an important determinant of social relations^26^. Spending more years in education therefore brings increased opportunities for not only developing cognitive skills, but also wider cultural awareness and social networks^4^. It is possible that this increased socialisation explains why females respond more positively to prolonged education than males.

Another possibility is that spending more years in education alters habits, practices, and health-related choices more favourably among females. Individuals genetically inclined to complete more years of schooling are more likely to engage in vigorous physical activity and less likely to engage in sedentary behaviour^3^. Educated females but not males have also been shown to be at a reduced risk of obesity^27^. Given the positive associations between BMI and wellbeing^15^, it is possible that sex differences in health behaviours contribute to the differential gains in the impact of education on wellbeing. Further research should attempt to understand these sex differences further to ensure more targeted support for males and females in schools. It is possible that males who remain in higher education would benefit from additional wellbeing support compared to females.

Our findings for intelligence add to the literature by providing causal evidence of the previously demonstrated negative associations with wellbeing^12,13^. In line with the current study, previous research also revealed a switch from positive to negative associations between intelligence and wellbeing after controlling for later-life outcomes like education attainment^12^. It has previously been suggested that this “residual” effect of intelligence on wellbeing may reflect the greater expectations of those high in intelligence with more education^12^. However, unlike previous observational research, the current findings were able to more directly rule out confounding of educational attainment to establish a causal and independent role for intelligence. The findings suggest it is possible that while educational attainment serves a protective function for those high in intelligence, the negative impact of lack of education is most detrimental for those with high intelligence. In other words, intelligence may negatively impact wellbeing among those who do not stay in education and who may be viewed as under-achievers.

The direct negative impact of intelligence on wellbeing may also reflect an underlying predisposition towards rumination and worry that is often reported among highly intelligent individuals^28^. It has been suggested that those high in intelligence have exaggerated physiological, neurological, and psychological responses to environmental stress that puts them at increased risk of mental health problems^29^. These reactions are more prevalent among those at the extreme end of the intelligence scale, which may explain why analyses using intelligence, but not educational attainment, produced negative associations with wellbeing. It is likely that such pupils may feel increased academic strain and pressure, and would benefit from additional wellbeing support at school.

It is also possible that different health behaviours underlie those high in intelligence compared to those who chose to stay on in higher education. Genetic studies of intelligence have revealed that unlike educational attainment, a genetic disposition towards higher intelligence is associated with reduced vigorous physical activity^3^. Intense physical activity is positively related to wellbeing across the lifespan^29,30^ and may therefore explain the positive association between education and wellbeing, and negative association between education and intelligence.

The finding that higher wellbeing positively predicts both intelligence and years of schooling aligns with previous research which has shown that adolescents with increased wellbeing tend to perform better academically^31^. By using a causal design, the current study reduces bias from reverse causality and confounding to provide support for improving wellbeing in schools^32^. The finding that wellbeing and educational attainment have a bidirectional relationship suggests that interventions aimed at improving wellbeing in schools could encourage further education and improved cognitive skills, and these in turn, could improve wellbeing in later life. Similarly, by keeping students engaged in school and increasing the likelihood of further education, wellbeing is likely to be improved, which could further increase the potential for higher education. Together these findings highlight their reciprocal relationship.

This study used both genetic and observational data to triangulate and provide further insight into associations between educational attainment, intelligence, and wellbeing. By using both univariable and multivariable MR, our study was able to investigate whether causal relations reflect direct or indirect effects. This is particularly important as the longer an individual spends in schooling, the greater their adult intelligence^33^. Thus, by using a multivariable design it was possible to separate such effects. Observational analyses were also adjusted for attrition and selective participation, helping to reduce the potential for bias. Some limitations of this study, however should be noted.

The first is that the MR analyses used GWAS data that included large samples from the UK Biobank^16^. Participants in the UK Biobank are generally more educated than the general population, which may have reduced the generalisability of the causal effect estimates. Given the cost of education in several of the studies contributing to the GWASs (that identify the genetic instrument), the effects of education may also be picking up socioeconomic effects. Previous MR studies on educational attainment have shown that after reweighting for sample selection, there is minimal impact of educational biases on the overall estimates^9^. Nevertheless, it is important that findings are interpreted in light of this potential selection bias, and that researchers are mindful of possible confounding by socioeconomic status.

The MR findings should also be interpreted in light of assortative mating and dynastic effects. Findings have shown that individuals are more likely to select a mate with a similar educational background^34^ and intelligence level^35^. This can lead to enriched educational or intelligence associated SNPs, as previously shown^36^, and may inflate subsequent MR estimates^37^. Dynastic effects can also bias MR estimates. Research has shown that parental educational level and family socioeconomic status predict the educational outcomes of their offspring^38^. Such dynastic effects as well as assortative mating can be investigated using a within-family design that adjusts for transmitted and non-transmitted alleles^39^. However, this was not possible in the current study as sufficient genotyped family data were not available. Nevertheless, a consistent result across MR estimates and observational analyses reduces the likelihood that MR estimates are confounding by characteristics that are transmitted across generations.

Further limitations of the current MR findings are that effect sizes relating to wellbeing are difficult to interpret. This is due to the nature of the meta-analytic findings which use multiple measures and phenotypes. While this is useful in testing whether or not there are possible causal effects, additional analyses using other methods are needed to estimate effect sizes. In addition, MR results using the intelligence GWAS from ref. ^40^. used UKBiobank samples that conditioned on socioeconomic status. Sensitivity analyses conducted after removing these samples produced consistent results but it is important that main analyses are interpreted with some caution.

The current observational findings should also be interpreted in light of some limitations. The only available information relating to educational attainment was whether or not individuals had at least a university degree. While detailed information has recently been collected on educational qualifications in ALSPAC for this age group, this data has not yet been released. Analyses were therefore unable to explore non-linear or cumulative effects of years of schooling, meaning it is not possible to ascertain whether a particular level of education confers an advantage or disadvantage for wellbeing. Such knowledge could have important implications for guiding and supporting students who continue their education to post-graduate level. Nevertheless, previous findings have shown that using years of education or a “Graduates versus non-graduates” proxy of education makes minimal difference to overall results^2^.

Other possible limitations are that wellbeing was assessed at 26 years, four years after the average person graduates from university. While research has shown that the gap in happiness between the educated and less educated widens as individuals age, this gap does not appear until around 35 years of age^41^. This is suggested to reflect a time in which uncertainties and student loan debt repayments may be reduced. Further longitudinal research should explore trajectories of mental health and wellbeing following completion of higher education to gain a more in-depth understanding of the long-term outcomes of education. This could also aid insight into differences noted between associations with educational attainment and either subjective happiness or life satisfaction.

Unlike subjective happiness, life satisfaction captures cognitive evaluations of one’s life. When reporting on life satisfaction, participants are therefore required to draw comparisons between their actual and desired life situation. It is possible that positive effects of educational attainment and intelligence on life satisfaction therefore reflect the fulfilment of years of hard work. Indeed, findings have shown that factors related to individual prosperity, including income and possessions, predict increased life satisfaction but not feelings of happiness^42^. Measures of subjective happiness do not require cognitive processing but capture immediate and accessible feelings of pleasure. Such feelings may be less influenced by the accumulation of factors gained from education and more influenced by immediate sensations like perceived general health. Young adults in the current study may have been transitioning into their new role in either employment, parenthood, or another life domain, and thus have been exposed to increased stress. This could have resulted in lower happiness levels at that time. Further investigation into the role of educational attainment on subjective happiness at earlier or later stages of life may lead to different estimates.

Overall, the findings from this study suggest important avenues for further research. While steps were taken to triangulate and improve the interpretation of the MR results, future research should consider using repeated measures of wellbeing to understand how causal effects may unfold over time. Research should also attempt to understand the factors underlying positive effects of educational attainment on wellbeing, and should consider additional mediating factors. This will be key to further dissecting the causal pathway and could reveal subtle differences between predictors of life satisfaction and subjective happiness^42^, and factors specific to wellbeing at specific life stages.

The degree to which educational attainment is driven by educational achievement (the grades you get) or other non-cognitive skills also requires further investigation. Unlike educational attainment, educational achievement is assessed using test and examination results. While highly correlated with cognitive ability^43^, educational outcomes reflect more than just intelligence^44,45^. These non-cognitive abilities, such as self-control, emotion regulation, grit and motivation, may explain why some remain in education where others do not, even if they do not excel academically or intellectually. Understanding more about the educational attainment phenotype and its drivers could yield important insight into why effects of educational attainment and intelligence may differ. This could have implications for both intervention and policy.

A final priority for further study is to ensure replication in other countries and among other ancestries. The average number of years spent in education differs worldwide^46^, and there exists significant global variability in wellbeing across sex^47^. Wellbeing has also changed over time, with some evidence to suggest population declines in subjective happiness^48^. The current observational findings are limited to individuals born between 1991 and 1992. Those following the typical education trajectory would therefore have graduated from university in 2012 or 2013. Research has shown that the time in which an individual graduates can predict wellbeing, with those graduating in times of higher unemployment more likely to have lower life satisfaction^49^. This needs to be accounted for when investigating more recent effects of educational attainment, particularly in light of the COVID-19 pandemic and on-going economic uncertainty. The pandemic caused significant distress to many due to unprecedented changes to economic situations and education systems. The implications of which for young people’s future education is not certain but remains a public health priority. Our findings add further weight to this and stress the importance of staying in school over and above cognitive abilities for good wellbeing.

To conclude, our findings demonstrate a unique benefit for wellbeing of staying in school, over and above improving cognitive abilities. Benefits are likely to be greater for females relative to males, suggesting other interventions may be necessary to improve the wellbeing of males who remain in education. The finding that intelligence has a direct negative impact on wellbeing suggests that students high in intelligence may be at risk of increased academic stress, and may therefore benefit from additional wellbeing support to alleviate these pressures. Schools aiming to improve student wellbeing more widely should focus less on improving cognitive abilities, and more on keeping students engaged in school.

## Methods

### Principles of Mendelian Randomisation

**Fig. 4:**
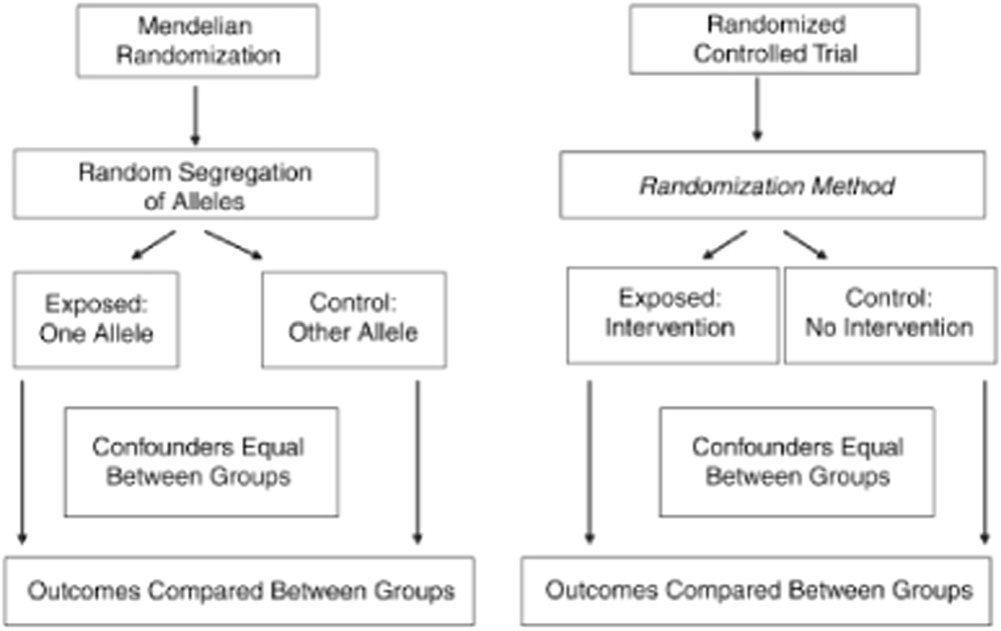
Principles of Mendelian Randomisation. Analogy between Mendelian randomisation (MR) and randomsed controlled trial (RCT).

Mendelian Randomisation (MR) is an instrumental variable method that uses natural genetic variation to study the causal effect of an exposure on an outcome^50,51^. The principles rely on Mendel’s law of segregation and independent assortment such that individuals inherit alleles that are independent of confounding traits and are randomly allocated at conception. Much like a Randomised Control Trial (RCT), the random segregation of participants, or alleles in the case of MR, are independent of any confounding variables, meaning confounding factors are assumed to be balanced across the two groups (see Fig. 4 taken from Davey Smith and Ebrahim^52^). Any differences that arise are therefore attributed to causal effects, providing that certain assumptions are met.

### Assumptions of MR

**Fig. 5:**
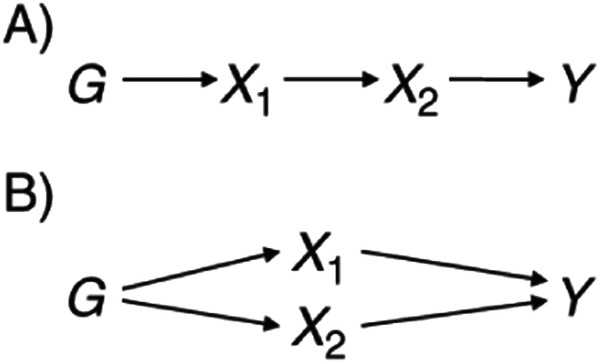
Pleiotropy within Mendelian Randomisation. A Directed Acyclic Graph (DAG) demonstrating vertical and horizontal pleiotropy in associations between an exposure and an outcome.

MR is based on the three key assumptions; (1) The instrument must be robustly associated with the exposure of interest; (2) The instrument must not be associated with factors that may confound the association between the exposure and the outcome; (3) If there is a causal effect of an exposure on an outcome, then genetic variants associated with the exposure should also predict the outcome, through the exposure only. If this last assumption is violated and genetic variants act on a second exposure that influences the outcome, this is known as pleiotropy. Some forms of pleiotropy, such as vertical pleiotropy, satisfy the principles of MR and do not inflict bias. This is because such pleiotropy occurs when genetic variants predict a primary and a secondary exposure which are both on the same causal pathway to the outcome (see Fig. 5a). This is the mechanism assumed in MR. If, however, the genetic variants act on the second exposure through a pathway other than through the primary exposure, this is known as horizontal pleiotropy (see Fig. 4b). This can lead to biased estimates in MR if not accounted for.

Many of the above assumptions rarely hold in MR, particularly where large numbers of genetic variants, whose functions are often unknown, are used as instruments^53^. This is because these can make pleiotropic pathways more likely. Fortunately, there are measures that can be taken to improve the reliability of MR, including running alternative versions of MR that make different assumptions about pleiotropy, as well as multivariable MR^54^.

### Multivariable MR

In traditional univariable MR, where the total effects of an exposure are investigated on an outcome, a second highly correlated exposure that is influenced by the same genetic variants would violate the assumptions of MR. An extension of MR, known as multivariable MR, allows exposures to be causally related provided the effects of the genetic variants are independent of the outcome^21^. Such an approach allows investigation into whether the two correlated exposures are causally related to the outcome, and whether such associations are independent of one another.

### Genetic data

To conduct an MR study, researchers must decide whether to use a one- or two-sample approach. Two sample MR requires two independent study samples, one is used to provide estimates for associations between genetic markers and the exposure, and the other for associations between genetic markers and the outcome. Benefit of using a two-sample approach include that it provides more power and pleiotropy sensitivity analyses. However, it comes with the additional assumption that the two samples represent separate participants from similar populations^3^. Genome wide association studies for this MR study were therefore carefully selected to ensure sample overlap was minimal.

Data for educational attainment was taken from the Years of Schooling GWAS^17^ This meta-analysed summary statistics from 64 samples, covering 15 different countries, all of European descent. Years of schooling were mapped and categorised across samples according to the 1997 International Standard Classification of Education (ISCED) scale^55^. This initial analysis identified 74 single nucleotide polymorphisms (SNPs) that were independently associated with years of schooling (*m* = 14.3, *SD* = 3.6) after adjustment for sex and ancestry principal components. A polygenic score constructed from the measured SNPs explained around 3.2% of the variance in educational attainment.

GWAS data were subsequently combined with those of 111,349 participants from the UK-Biobank (UKB)^17^. This replication resulted in a GWAS sample of 405,072 participants, and increased the number of associated genetic variants from 74 to 162. The current study, however, used data from the original discovery GWAS as opposed to the larger replication to reduce sample overlap (from 34% to 9%). Analyses were repeated using the larger replication cohort to ensure consistency (see Supplementary Table 14). It is important to note that while a larger and more recent meta-GWAS is available for educational attainment^56^, these samples largely overlap with those of the wellbeing GWAS used in the present study. Sensitivity analyses using MRLap, which is a method that accounts for potential sample overlap^57^, suggested estimates for univariable MR were similar to those using SNP estimates including23andMe (see Supplementary Table 15).

For intelligence, data were derived from the largest GWAS of intelligence to date (*n* = 269,867)^40^. This study was based on 14 cohorts that assessed intelligence using various neurocognitive tests of logical, verbal, spatial, and technical ability. Despite the different assessments, all cohorts extracted a single sum, mean, or factor score which was used to index general intelligence. Correlations across cohort measures were on average 0.67. Overall, the GWAS identified 242 lead SNPs associated with intelligence at genome-wide significance. Polygenic scores derived from these SNPs explained up to 5.2% of the variance in intelligence in four independent samples^40^.

Wellbeing data were taken from a multivariate genome-wide-association meta-analysis (GWAMA) of wellbeing^16^. This used the widely documented genetic overlap between four traits, life satisfaction, positive affect, depression, and neuroticism, to identify genetic variants associated with wellbeing. Two novel and complementary methods: An N-weighted multivariate GWAMA (N-GWAMA) and a model-averaging GWAMA (MA-GWAMA) approach were used. The N-GWAMA investigated a unitary effect of all traits, referred to collectively as the wellbeing spectrum, while MA-GWAMA relaxed the assumption of a unitary effect to study trait-specific estimates. Findings from the N-GWAMA revealed 231 independent SNPs associated with the wellbeing spectrum, while the MA-GWAMA resulted in 148 independent loci for life satisfaction and 191 for positive affect. The incremental R^2^ for these SNPs was slightly lower than those derived from the N-GWAMA, therefore the current study used estimates related to the wellbeing spectrum. In particular, polygenic scores constructed from the N-GWAMA and MA-GWAMA explained 0.94% and 0.92% of the variance in life satisfaction and 1.10% and 1.06 of the variance in positive affect. Follow-up analyses were carried out to explore specific estimates for the individual wellbeing components (Supplementary Tables 5 to 8).

Approximately 11% of the individuals from the wellbeing GWAS sample were also included in the educational attainment GWAS sample^17^, and around 8% in the intelligence GWAS sample^40^. This overlap is similar to previous MR studies investigating wellbeing^15^.

### Genetic instrument construction

Genetic variants included were those that passed the genome-wide level of significance (*p* < 5 × 10^−8^) and were independent. Clumping was performed to ensure independence at r 2 < 0.001 within an 10,000 kb window. Data harmonisation was then performed using the TwoSampleMR package^58^, where allele frequencies were used to align palindromic SNPs down to a minor allele frequency of 0.42. For univariable MR analyses, instrument strength was calculated using an F statistic greater than 10^59^. For multivariable analyses, the Sanderson–Windmeijer partial F-statistic was used^60^.

Following data harmonisation, analyses exploring causal effects of educational attainment on intelligence used a total of 63 SNPs (*F* = 38.44), while analyses exploring the impact of intelligence on educational attainment used 144 SNPs (*F* = 42.72). Note that an F greater than 10 indicates the analysis is unlikely to suffer from bias due to a weak instrument^61^.

Analyses exploring the total causal effects of educational attainment on wellbeing used 54 SNPs that were available following data harmonisation (*F* = 38.88). For analyses exploring total causal effects of wellbeing on educational attainment, there were 147 SNPs available following data harmonisation (*F* = 40.78). Of these, 90 SNPs (61.2%) formed part of the original 232 SNPs identified in the wellbeing GWAS.

Analyses testing causal effects of intelligence on wellbeing used 126 SNPs following data harmonisation (*F* = 43.35). Analyses testing causal effects of wellbeing on intelligence used 128 SNPs (*F* = 40.83), of which 71 (55.4%) formed part of the original 232 lead SNPs in wellbeing GWAS.

As per the univariable MR analyses, variants were selected for the multivariable MR if they passed the genome-wide level of significance (*p* < 0.001 and 10,000 kb were used as conditions of clumping), and palindromic SNPs were aligned using a minor allele frequency of 0.42. Note that SNPS selected for multivariable MR are the exposure SNPs that are associated with the outcome, conditional on the other exposure. This resulted in 151 SNPs available for the multivariable MR analysis, a full list of which can be found in the Supplementary Table 14.

### Statistical analyses

#### Univariable MR

Univariable MR analyses were conducted using the TwoSampleMR package, version 0.5.6 in R^61^. These analyses were used to test for causal associations between educational attainment and intelligence, and between wellbeing and the two exposures: educational attainment and intelligence. All univariable analyses were run using four different versions of two-sample MR. The inverse variance weighted (IVW) method was used as the main analysis as this assumes no directional pleiotropy, with sensitivity analyses including mendelian randomisation-Egger (MR-Egger), weighted median, and weighted mode. These have each been described in detail elsewhere^62,63^, but were included here as each makes a different assumption about pleiotropy. A consistent effect across the different methods can therefore provide more confidence that the assumptions are valid. In addition, a simulation extrapolation (SIMEX) correction was applied to MR-Egger estimates to correct coefficients where regression dilution was lower than 0.9^54^. A consistent result across these provides further support for a true causal effect.

To further assess the robustness of the results, heterogeneity was estimated using Cochran’s Q^64^. Tests of heterogeneity reveal how consistent the causal estimate is across SNPs, which can be used as an indicator of pleiotropy. Based on previous findings, it was anticipated that heterogeneity would be high^11^. High pleiotropy will only impose bias if it is directional and horizontal, and therefore the MR Egger intercept was used to check for evidence of directional/horizontal pleiotropy. A multiplicative random effects IVW regression was also chosen to adjust for this. Steiger filtering was conducted where more than one SNP explained more of the variance in the outcome than the exposure, which could suggest possible reverse causation.

As a sensitivity check, we repeated analyses using the intelligence GWAS without the UKB. This was because samples using the UKB conditioned on socioeconomic status.

### Multivariable MR

Multivariable MR was then used to estimate the direct effects of educational attainment and intelligence on wellbeing, independent of the other. We also performed two additional multivariable MR analyses: (1) intelligence and well-being on years of schooling; (2) years of schooling and well-being on intelligence. All analyses were run using the MVMR package^21^ and the MendelianRandomization package (Rees et al., 2017) in R. As per the univariable analyses, heterogeneity was checked using Cochran’s Q, and conditional F statistics using the Sanderson–Windmeijer partial F-statistic^60^.

### Observational analyses

#### Sample

Observational data were taken from the Avon Longitudinal Study of Parents and Children (ALSPAC^24^) a prospective cohort study based in the United Kingdom. Pregnant women residing in the former Avon area were enrolled if they had an expected delivery date between April 1991 and December 1992^65^. The initial cohort consisted of 14,062 live births but has since increased to 14,901 children following further recruitment^66^. Data gathered from 22 years and onwards were collected and managed using REDCap electronic data capture tools hosted at the University of Bristol^67^. REDCap (Research Electronic Data Capture) is a secure, web-based software platform designed to support data capture for research studies. Please note that the study website contains details of all the data that is available through a fully searchable data dictionary and variable search tool (http://www.bristol.ac.uk/alspac/researchers/our-data/).

Participants included in the current study were those who completed a measure of educational attainment at age 26, an intelligence assessment at age 8, as well as relevant wellbeing measures at age 26 (see Supplementary Tables 10, 15, and 16 for further information about the measures, and Supplementary Fig. 4 for a flowchart of data availability). In total, there were 2844 participants with complete data on intelligence, wellbeing, and educational attainment. The wellbeing of participants with complete data on either intelligence (*n* = 3179) or educational attainment (*n* = 3788) did not differ (see Supplementary Table 9), therefore analyses were conducted on the two predictors separately to maximise power.

Ethical approval for the ALSPAC study was obtained from ALSPAC Ethics and Law Committee and the Local Research Ethics Committees. Informed consent for the use of data collected via questionnaires and clinics was obtained from participants following the recommendations of the ALSPAC Ethics and Law Committee at the time.

### Measures

Educational attainment was based on university degree completion. Participants responded to the item, ‘Do you have a university degree?’ which was included in the Life@26 questionnaire sent to 9230 (66%) participants in ALSPAC. While detailed information was collected on educational qualifications in ALSPAC for this age group, this data has not yet been released.

In total, 4029 completed the questionnaire, reflecting a 43.7% response rate. Answers included ‘yes’ (*n* = 2452), ‘no’ (*n* = 1377) or ‘still at university’ (*n* = 200). Those who responded ‘still at university’ were excluded from analyses. This is because individuals at university at 26 years would not necessarily represent those who followed the typical educational trajectory. For example, individuals may have taken a break from education and returned, or may be re-taking courses. Including such individuals may therefore have skewed analyses or created noise between the observational findings and those from the MR, which were based on years of schooling. Thus those with the highest number of total years would reflect those who earned a PhD degree at university. This could not be guaranteed among the current cohort of individual’s still studying due to the unavailability of further information.

Intelligence was assessed at the Focus at 8 clinic using the Wechsler Intelligence Scale for Children (WISC-III^68^). The WISC comprises of ten subtests, including five verbal tests and five performance tests, as well as a forwards/backwards digit span test. The overall continuous score represents the total scaled scores across verbal and performance tests which were calculated using the WISC manual.

Wellbeing was captured at 26 years using the Subjective Happiness Scale^69^, the Satisfaction with Life Scale^70^, and the Meaning in Life Scale^71^. The current study focused on the Subjective Happiness Scale and the Satisfaction with Life Scale to ensure a close replication of the MR study. The Subjective Happiness Scale includes 4 items, with overall scores reflective of greater subjective happiness. The scale has high internal consistency and test-retest reliability, and is suitable for different age, occupational, and cultural groups^69^. The Satisfaction with Life Scale is a 5-item measure that was designed to capture cognitive judgments of one’s life satisfaction as opposed to positive affect^70^. Answers are coded so that a higher overall score reflects greater life satisfaction. Correlations between life satisfaction and subjective happiness were *r* = 0.65. Both wellbeing measures were z-standardised to facilitate comparisons between the two.

### Statistical analyses

In an attempt to first replicate the MR findings, separate linear regression models were first run. These investigated associations between educational attainment and wellbeing, and between intelligence and wellbeing. Wellbeing was assessed using subjective happiness and life satisfaction, with each ran as a separate regression. Analyses were repeated after including an interaction between sex and the predictor to test for possible sex differences.

All linear models were corrected for multiple testing using Benjamini Hochberg False Discovery Rate (FDR^22^). This was based on a total of 62 tests to include models adjusted for attrition and missing data.

As educational attainment was recorded using a binary response, analyses checking for possible non-linearity were conducted for intelligence only. Models investigating associations between intelligence and wellbeing included either a quadratic, cubic, or quartic polynomial term, as per previous research focused on mental health in young adulthood^72^. Additional analyses also explored non-linearity using spline regressions. This is because polynomial terms may not be flexible enough to capture the relationship between intelligence and wellbeing as they impose a global structure on all of the data. Spline regressions were therefore included within a Generalised Additive Model (GAM) which was run using the ‘mgcv’ R package^73^. The model of best fit was determined using the Akaike information criterion (AIC) and Bayesian information criterion (BIC), as previously recommended^74^.

To further investigate possible factors driving associations with wellbeing, a linear model was run with an interaction term between the two predictors (educational attainment*intelligence). This was used to provide insight into the extent to which the relationship between intelligence and wellbeing is moderated by educational attainment and vice versa. Two interaction models were run, one predicting subjective happiness and one predicting life satisfaction. Finally, to test if any associations were explained by income, as noted in previous studies^2^, we repeated analyses after adjustment for family income.

The impact of attrition in the observational analyses was investigated using inverse probability weighting (IPW) and multiple imputation, as per previous studies using ALSPAC^75^. Multiple imputation was conducted using the Chained Equations (MICE) package^76^. Based on Rubin’s rules^77^, 60 imputations were conducted. The variables selected to impute data have been previously associated with missingness in ALSPAC and can be found in Supplementary Table 16. It was important that analyses accounted for missing data as there was some evidence to suggest selective attrition (see Supplementary Table 15).

### Supplementary information

Supplementary Information

## Data Availability

All data sources used for the MR SNP-exposure and SNP-outcome associations are publicly available. Summary data from the Okbay et al. ^17^ Years of Schooling GWAS were downloaded from the SSGAC website SSGAC Login (thessgac.com), and the summary data for the intelligence GWAS^40^ were obtained from the CNCR website GWAS Summary Statistics | CTG (cncr.nl). Summary statistics for the wellbeing GWAS, excluding results from 23AndMe cohort, were downloaded from https://surfdrive.surf.nl/files/index.php/s/Ow1qCDpFT421ZOO The observational ALSPAC data used in this study is not publicly available because the informed consent does not allow data to be made freely available through any third party maintained public repository. Data used for this submission, however, can be made available on request to the ALSPAC Executive. Please refer to the ALSPAC data management plan which describes the policy regarding data sharing. This is through a system of managed open access. Full instructions for applying for data access can be found here: http://www.bristol.ac.uk/alspac/researchers/access/. The ALSPAC study website contains details of all the data that are available (http://www.bristol.ac.uk/alspac/researchers/our-data/), and a comprehensive list of grants funding is also available on the ALSPAC website (http://www.bristol.ac.uk/alspac/external/documents/grant-acknowledgements.pdf).

## References

[1] Oreopoulos P, Salvanes KG (2011). Priceless: the nonpecuniary benefits of schooling. J. Econ. Perspect..

[2] Powdthavee N, Lekfuangfu WN, Wooden M (2015). What’s the good of education on our overall quality of life? A simultaneous equation model of education and life satisfaction for Australia. J. Behav. Exp. Econ..

[3] Davies NM (2019). Multivariable two-sample Mendelian randomization estimates of the effects of intelligence and education on health. eLife.

[4] Yuan S, Xiong Y, Michaëlsson M, Michaëlsson K, Larsson SC (2021). Genetically predicted education attainment in relation to somatic and mental health. Sci. Rep..

[5] O’Donnell, G., Deaton, A., Durand, M., Halpern, D., & Layard, R. *Wellbeing and policy* (Legatum Institute: London, 2014).

[6] Diener E (2000). Subjective well-being. The science of happiness and a proposal for a national index. Am. Psychol..

[7] Cuñado J, Pérez de Gracia F (2012). Does education affect happiness? Evidence for Spain. Soc. Indic. Res..

[8] Nikolaev B (2018). Does higher education increase hedonic and eudaimonic happiness?. J. Happiness Stud..

[9] Davies, N. M., Dickson, M., Davey Smith, G., Windmeijer, F., & van den Berg, G. J. The causal effects of education on adult mortality, health, and income: evidence from Mendelian randomization and the raising of the school leaving age. *Int. J. Epidemiol.**dyad104*, 10.1093/ije/dyad104 (2023). 10.1093/ije/dyad104 PMC10749779 37463867

[10] Colom R, Karama S, Jung RE, Haier RJ (2010). Human intelligence and brain networks. Dialogues Clin. Neurosci..

[11] Anderson EL (2020). Education, intelligence and Alzheimer’s disease: evidence from a multivariable two-sample Mendelian randomization study. Int. J. Epidemiol..

[12] Clark AE, Lee T (2021). Early-life correlates of later-life wellbeing: evidence from the Wisconsin Longitudinal Study. J. Econ. Behav. Organ..

[13] Flèche S, Lekfuangfu WN, Clark AE (2021). The long-lasting effects of family and childhood on adult wellbeing: evidence from British cohort data. J. Econ. Behav. Organ..

[14] Davey Smith G, Hemani G (2014). Mendelian randomization: genetic anchors for causal inference in epidemiological studies. Hum. Mol. Genet..

[15] Wootton RE (2018). Evaluation of the causal effects between subjective wellbeing and cardiometabolic health: mendelian randomisation study. BMJ.

[16] Baselmans BML (2019). Multivariate genome-wide analyses of the well-being spectrum. Nat. Genet..

[17] Okbay A (2016). Genetic variants associated with subjective well-being, depressive symptoms, and neuroticism identified through genome-wide analyses. Nat. Genet..

[18] Lyubomirsky S, King LA, Diener E (2005). The benefits of frequent positive affect: does happiness lead to success?. Psychol. Bull..

[19] Salinas-Jiménez MM, Artés J, Salinas-Jiménez J (2013). How do educational attainment and occupational and wage-earner statuses affect life satisfaction? A gender perspective study. J. Happiness Stud..

[20] Major JT, Johnson W, Deary IJ (2014). Linear and nonlinear associations between general intelligence and personality in project TALENT. J. Personal. Soc. Psychol..

[21] Sanderson E, Davey Smith G, Windmeijer F, Bowden F (2019). An examination of multivariable Mendelian randomization in the single-sample and two-sample summary data settings. Int. J. Epidemiol..

[22] Benjamini Y, Hochberg Y (1995). Controlling the false discovery rate: a practical and powerful approach to multiple testing. J. R. Stat. Soc. Ser. B (Methodol.).

[23] Adams CD (2020). A multivariable Mendelian randomization to appraise the pleiotropy between intelligence, education, and bipolar disorder in relation to schizophrenia. Sci. Rep..

[24] Boyd A (2013). Cohort profile: the ‘children of the 90s’—the index offspring of the avon longitudinal study of parents and children. Int. J. Epidemiol..

[25] Kroll C (2011). Different things make different people happy: examining social capital and subjective well-being by gender and parental status. Soc. Indic. Res..

[26] Witkow MR, Fuligni AJ (2010). In-school versus out-of-school friendships and academic achievement among an ethnically diverse sample of adolescents. J. Res. Adolesc..

[27] Amin, V., Behrman, J. R., & Spector, T. D. Does More Schooling Improve Health Outcomes and Health Related Behaviors? Evidence from U.K. Twins. *Econ. Educ. Rev.* **35**. 10.1016/j.econedurev.2013.04.004 (2013). 10.1016/j.econedurev.2013.04.004 PMC3885175 24415826

[28] Karpinski RI, Kolb AMK, Tetreault NA, Borowski TB (2018). High intelligence: a risk factor for psychological and physiological overexcitabilities. Intelligence.

[29] Costigan SA, Lubans DR, Lonsdale C, Sanders T, del Pozo Cruz B (2019). Associations between physical activity intensity and well-being in adolescents. Prevent. Med..

[30] Ku P-W, Fox KR, Liao Y, Sun W-Y, Chen L-J (2016). Prospective associations of objectively assessed physical activity at different intensities with subjective well-being in older adults. Qual. Life Res..

[31] Kaya M, Erdem C (2021). Students’ well-being and academic achievement: a meta-analysis study. Child Indic. Res..

[32] Bonell C (2014). Why schools should promote students’ health and wellbeing. BMJ.

[33] Ritchie SJ, Tucker-Drob EM (2018). How much does education improve intelligence? A Meta-Analysis. Psychol. Sci..

[34] Domingue BW, Lie H, Okbay A, Belsky DW (2017). Genetic heterogeneity in depressive symptoms following the death of a spouse: polygenic score analysis of the US Health and Retirement Study. Am. J. Psychiatry.

[35] Plomin R, Deary I (2015). Genetics and intelligence differences: five special findings. Mol. Psychiatry.

[36] Torvik FA (2022). Modeling assortative mating and genetic similarities between partners, siblings, and in-laws. Nat. Commun..

[37] Hartwig FP, Davies NM, Davey Smith G (2018). Bias in Mendelian randomization due to assortative mating. Genet. Epidemiol..

[38] Wang, B. et al. Genetic nurture effects on education: a systematic review and meta-analysis. *bioRxiv*. 10.1101/2021.01.15.426782 (2021). 10.1016/j.ajhg.2021.07.010 PMC8456157 34416156

[39] Munafò MR, Davies NM, Davey Smith G (2019). Can genetics reveal the causes and consequences of educational attainment?. J. R. Stat. Soc., Ser. A.

[40] Savage JE (2018). Genome-wide association meta-analysis in 269,867 individuals identifies new genetic and functional links to intelligence. Nat. Genet..

[41] Nikolaev B, Rusakov P (2015). Education and happiness: an alternative hypothesis. Appl. Econ. Lett..

[42] Diener E, Ng W, Harter J, Arora R (2010). Wealth and happiness across the world: material prosperity predicts life evaluation, whereas psychosocial prosperity predicts positive feeling. J. Personal. Soc. Psychol..

[43] Deary IJ, Strand S, Smith P, Fernandes C (2007). Intelligence and educational achievement. Intelligence.

[44] Krapohl E (2014). The high heritability of educational achievement reflects many genetically influenced traits, not just intelligence. Proc. Natl. Acad. Sci..

[45] Demange PA (2021). Investigating the genetic architecture of noncognitive skills using GWAS-by-subtraction. Nat. Genet..

[46] Lee JJ (2018). Gene discovery and polygenic prediction from a genome -wide association study of educational attainment in 1.1 million individuals. Nat. Genet..

[47] Ruggeri K, Garcia-Garzon E, Maguire Á, Matz S, Huppert FA (2020). Well-being is more than happiness and life satisfaction: a multidimensional analysis of 21 countries. Health Qual. Life Outcomes.

[48] Jebb AT, Morrison M, Tay L, Diener E (2020). Subjective well-being around the world: trends and predictors across the Life Span. Psychol. Sci..

[49] Cutler DM, Huang W, Llera-Muney A (2015). When does education matter? The protective effect of education for cohorts graduating in bad times. Soc. Sci. Med..

[50] Davey Smith G, Hemani G (2014). Mendelian randomization: genetic anchors for causal inference in epidemiological studies. Hum. Mol. Genet.

[51] Davies NM, Holmes MV, Davey Smith G (2018). Reading Mendelian randomisation studies: a guide, glossary, and checklist for clinicians. BMJ.

[52] Davey Smith, G., Ebrahim, S. Mendelian Randomization: Genetic Variants as Instruments for Strengthening Causal Inference in Observational Studies. In: Weinstein M., Vaupel J. W., Wachter K. W., (Eds). National Research Council (US) Committee on Advances in Collecting and Utilizing Biological Indicators and Genetic Information in Social Science Surveys; Biosocial Surveys, (336-366). Washington (DC): National Academies Press. (2008). 21977539

[53] Bowden J (2016). Assessing the suitability of summary data for two-sample Mendelian randomization analyses using MR-Egger regression: the role of the I2 statistic. Int. J. Epidemiol..

[54] Hemani G, Bowden J, Davey Smith G (2018). Evaluating the potential role of pleiotropy in Mendelian randomization studies. Hum. Mol. Genet..

[55] UNESCO. International standard classification of education—ISCED 1997. In *Advances in Cross-National Comparison* (eds Hoffmeyer-Zlotnik, J.H.P. & Wolf, C.) (2003, Springer, Boston, MA). http://www.uis.unesco.org/Library/Documents/isced97-en.pdf

[56] Okbay A (2022). Polygenic prediction of educational attainment within and between families from genome-wide association analyses in 3 million individuals. Nat. Genet..

[57] Mounier N, Kutalik Z (2023). Bias correction for inverse variance weighting Mendelian randomization. Genet. Epidemiol..

[58] Hemani G (2018). The MR-Base platform supports systematic causal inference across the human phenome. eLife.

[59] Staiger D, Stock JH (1997). Instrumental variables regression with weak instruments. Econometrica.

[60] Sanderson E, Windmeijer F (2016). A weak instrument F-test in linear IV models with multiple endogenous variables. J. Econ..

[61] R Core Team. R: A language and environment for statistical computing. R Foundation for Statistical Computing, Vienna, Austria. https://www.R-project.org/ (2021).

[62] Bowden J, Davey Smith G, Burgess S (2015). Mendelian randomization with invalid instruments: effect estimation and bias detection through Egger regression. Int. J. Epidemiol..

[63] Burgess S (2019). Guidelines for performing Mendelian randomization investigations. Wellcome Open Res..

[64] Bowden J (2018). Improving the visualization, interpretation and analysis of two-sample summary data Mendelian randomization via the Radial plot and Radial regression. Int. J. Epidemiol..

[65] Fraser A (2013). Cohort profile: the avon longitudinal study of parents and children: ALSPAC mothers cohort. Int. J. Epidemiol..

[66] Northstone K (2019). The Avon Longitudinal Study of Parents and Children (ALSPAC): an update on the enrolled sample of index children in 2019. Wellcome Open Res..

[67] Harris PA (2009). Research electronic data capture (REDCap)—A metadata-driven methodology and workflow process for providing translational research informatics support. J. Biomed. Inform..

[68] Wechsler, D., Golombok, J., & Rust, J. WISC-III UK Wechsler intelligence scale for children: UK manual. The Psychological Corporation; Sidcup, UK. (1992).

[69] Lyubomirsky S, Lepper H (1999). A measure of subjective happiness: preliminary reliability and construct validation. Soc. Indic. Res..

[70] Diener E, Emmons RA, Larsen RJ, Griffin S (1985). The satisfaction with life scale. J. Personal. Assess..

[71] Steger MF, Frazier P, Oishi S, Kaler M (2006). The meaning in life questionnaire: assessing the presence of and search for meaning in life. J. Counseling Psychol..

[72] Kwong A (2019). Identifying critical points of trajectories of depressive symptoms from childhood to young adulthood. J. Youth Adolesc..

[73] Wood, S. *Generalized additive models: an introduction with R* CRC Press: Florida, 2006).

[74] Singer J. D., & Willett, J. B. *Applied Longitudinal Data Analysis: Modelling Change and Event Occurence* (Oxford University Press: New York, 2003).

[75] Cornish RP, Tilling K, Boyd A, Davies A, Macleod J (2015). Using linked educational attainment data to reduce bias due to missing outcome data in estimates of the association between the duration of breastfeeding and IQ at 15 years. Int. J. Epidemiol..

[76] Van Buuren S, Groothuis-Oudshoorn K (2010). mice: Multivariate imputation by chained equations in R. J. Stat. Software.

[77] Little, R. J., & Rubin, D. B. *Statistical Analysis with Missing Data*. (John Wiley & Sons, Hoboken, 2014).

