# Supplementary material for: An exploration into the causal relationships between educational attainment, intelligence, and wellbeing: an observational and two-sample Mendelian randomisation study
Source: Npj Ment Health Res. 2024 May 9;3:23. doi: 10.1038/s44184-024-00066-x (PMC11082190; doi:10.1038/s44184-024-00066-x)
Supplement: Supplementary file 1 — Supplementary Information [file 44184_2024_66_MOESM1_ESM.pdf]

An exploration into the causal relationships between educational attainment, intelligence, and wellbeing: an observational and two-sample Mendelian randomisation study

Supplementary Methods

GWAS information

Educational attainment

Data for educational attainment was taken from the GWAS of 293,723 participants (Okbay et al., 2016). This study meta-analysed summary statistics from 64 samples spanning 15 different countries. All subjects were of European descent and reported their educational attainment when they were aged 30 or above. The reported qualifications were categorised into one of seven bands based on the 1997 International Standard Classification of Education (ISCED) scale (UNESCO, 2006). These categories were then used to map the equivalent number of years in schooling. Findings from the initial cohort identified 74 independent genetic variants associated with years of schooling (mean=14.3, SD= 3.6). In subsequent analyses, data were combined with those of 111,349 participants from the UK-Biobank to increase the number of associated independent SNPs from 74 to 162.

Sample overlap between participants in either the education attainment discovery, or discovery and replication cohort was 8.6% with the intelligence GWAS. The intelligence GWAS used the second release of UKB participants and therefore sample overlap with the interim release included in the educational attainment GWAS was minimal.

Intelligence

For intelligence, data were derived from the largest GWAS of intelligence to date (n = 269,867) (Savage et al.,

2018). This meta-analysis of 14 cohorts identified 242 lead SNPs at genome-wide significance, located across 213 independent genomic risk loci (after merging regions <250 KB apart, r^2=0.1). Various neurocognitive tests of logical, verbal, spatial, and technical ability were included, such as the forward and backward digit span, however, genetic correlations between cohorts were considerable (mean = 0.67). There was also no evidence to suggest heterogeneity across cohorts in the SNP associations. Approximately 3.5% of the participants from the intelligence GWAS were included in the educational attainment cohorts.

Wellbeing

Wellbeing data was taken from the multivariate genome-wide-association meta-analysis (GWAMA)

(Baselmans, Jansen, Ip, van Dongen, Abdellaoui, van de Weijer & Bao et al., 2019). This study used the widely documented genetic overlap between four traits, positive affect, life satisfaction, depression, and neuroticism, to run two novel methods: An N-weighted multivariate GWAMA (N-GWAMA) and a model-averaging GWAMA (MA-GWAMA). The N-GWAMA was used to identify a unitary effect of the SNPs on all traits, which they collectively refer to as the wellbeing spectrum. For these analyses, the estimated SNP effects on neuroticism and depressive symptoms were reversed. The authors identified 231 independent loci associated with the wellbeing spectrum. For the MA-

GWAMA, the unitary effect assumption was relaxed, and instead trait-specific estimates were generated for each SNP. This resulted in 148 independent loci for life satisfaction, 191 for positive affect, 263 for neuroticism, and 239 for depressive symptoms. N-GWAMA and MA-GWAMA are complementary methods that can be used together. We therefore explore associations using data from both, taking the N-GWAMA estimates of the wellbeing spectrum first, and then exploring specific estimates for positive affect and life satisfaction in follow-up analyses.

Sample overlap between participants from the wellbeing GWAS and those included in the educational attainment discovery GWAS is approximately 11.3%. This increases to approximately 16.1% for the educational attainment discovery and replication GWAS. Sample overlap between participants included in the wellbeing and intelligence GWAS is estimated to be 8.3%.

Analyses using 405k educational attainment GWAS.

For these analyses, a total of 162 independent SNPs were available for educational attainment, of which 152 were identified in the intelligence GWAS. Following data harmonisation, 59 SNPs remained. For analyses exploring possible casual effects of intelligence on educational attainment, there were 210 SNPs available in both GWAS, of which 186 SNPs were available after 24 palindromic SNPs were removed during data harmonisation.

Results

Univariable MR testing causal associations between educational attainment and wellbeing

While univariable analyses did not replicate using MR-Egger, this is unlikely to be a result of directional pleiotropy. Directional pleiotropy is accounted for in MR-Egger, therefore if directional pleiotropy is present, it can result in the removal of any causal effects. This seems unlikely based on the current findings as the MREgger intercept did not differ from zero. The funnel plots also provided evidence of balanced pleiotropy and there was no evidence in the forest plots to suggest that associations were strongly driven by one singlenucleotide polymorphisms (SNP) (see Supplementary Figures 1 and 2). These combined findings suggest that associations are unlikely to be significantly driven by directional pleiotropy. It is also important to note that Steiger filtering revealed that all educational attainment SNPs were more associated with educational attainment than wellbeing, and only 4 out of a total 147 wellbeing SNPs (2.7%) explained more of the variance in educational attainment than wellbeing. Analyses repeated after removing these SNPs revealed consistent results (see Supplementary Table 3), suggesting minimal bias from reverse causation. It is therefore likely that findings reflect measurement error, as evaluated by the low regression dilution I2 statistic (see Supplementary Table 2). Only 4 out of a total 147 wellbeing SNPs (2.7%) explained more of the variance in educational attainment than wellbeing. Analyses were repeated after removing these SNPs and results were largely consistent (see Supplementary Table3).

Supplementary tables and figures

Index of Supplementary Tables

- **Supplementary Table 1:** Univariable MR analyses assessing bidirectional associations between educational attainment and intelligence
- **Supplementary Table 2**: Univariable MR assessing bidirectional associations between educational attainment and wellbeing, and between intelligence and wellbeing
- **Supplementary Table 3:** Univariable MR analyses assessing associations between wellbeing and educational attainment, and between wellbeing and intelligence following Steiger filtering
- **Supplementary Table 4:** F statistic and regression dilution I^2 statistic for the heterogeneity of SNP-exposure effects
- **Supplementary Table 5:** Univariable MR analyses using intelligence GWAS without UKB
- **Supplementary Table 6:** Univariable MR analyses assessing impact of educational attainment and intelligence on positive affect
- **Supplementary Table 7:** Univariable MR analyses assessing impact of educational attainment and intelligence on life satisfaction
- **Supplementary Table 8:** Univariable MR analyses assessing impact of educational attainment and intelligence on depressive symptoms
- **Supplementary Table 9:** Univariable MR analyses assessing impact of educational attainment and intelligence on neuroticism
- **Supplementary Table 10**: Comparison of main variable scores between ALSPAC subsamples
- **Supplementary Table 11:** Description of main study variables in ALSPAC
- **Supplementary Table 12:** Observational regression results assessing linear associations between educational attainment and wellbeing, and between intelligence and wellbeing (unstandardised)
- **Supplementary Table 13:** Observational regression results assessing linear associations between educational attainment and wellbeing, and between intelligence and wellbeing with adjustments for family income
- **Supplementary Table 14:** Observational regression results from linear, quadratic, cubic and quartic models assessing associations between intelligence and wellbeing
- **Supplementary Table 15:** Univariable MR analyses assessing bidirectional associations between educational attainment (using discovery and replication cohort)
- **Supplementary Table 16:** Results from MRLap tests of impact of sample overlap
- **Supplementary Table 17:** List of SNPs used for multivariable MR
- **Supplementary Table 18:** Selective attrition for educational attainment and intelligence based
- **Supplementary Table 19:** Variables included in multiple imputation

Index of Supplementary Figures

- **Supplementary Figure 1:** Funnel plots for univariate MR analysis of years of schooling on wellbeing and wellbeing on years of schooling.
- **Supplementary Figure 2:** Forest plots from univariate MR analysis of years of schooling on wellbeing and wellbeing on years of schooling.
- **Supplementary Figure 3:** Funnel and forest plots from univariate MR analysis of wellbeing on intelligence.
- **Supplementary Figure 4**: Flowchart of data available for use in ALSPAC.

Supplementary Table 1: Univariable MR analyses assessing bidirectional associations between educational attainment and intelligence

		Causal effect estimates		Heterogeneity statistics		
	N SNPs	β (95% CI)	P	Q	df	P
**Years of schooling on intelligence**						
IVW	63	0.736 (0.647, 0.826)	4.88E-58	272.2	62	8.90E-26
MR-Egger	63	1.171 (0.724, 1.62)	3.12E-06	256.3	61	3.95E-28
MR-Egger intercept	63	-0.008 (-0.016, 0.002)	0.056	-	-	-
MR-Egger (SIMEX) [a]	63	1.850 (1.28, 2.42)	1.44E-08	-	-	-
Weighted median	63	0.655 (0.566, 0.743)	1.20E-50	-	-	-
Weighted mode	63	0.632 (0.410, 0.854)	1.25E-07	-	-	-
**Intelligence on years of schooling**						
IVW	144	0.398 (0.359, 0.438)	2.40E-85	466.1	143	7.11E-36
MR-Egger estimate	144	0.534 (0.346, 0.721)	1.17E-07	459.9	142	4.16E-35
MR-Egger intercept	144	-0.003 (-0.006, 0.001)	0.150	-	-	-
MR-Egger (SIMEX)	144	0.815 (0.562, 1.07)	2.69E-09	-	-	-
Weighted median	144	0.341 (0.301, 0.381)	1.92E-65	-	-	-
Weighted mode	144	0.277 (0.175, 0.379)	1.56E-06	-	-	-

Note: IVW= Inverse variance weighted estimate. Analyses conducted using the educational attainment discovery cohort (*n*=293,723).
[a] Weighted simulation extrapolation (SIMEX) correction applied.

Supplementary Table 2: Univariable MR assessing bidirectional associations between educational attainment and wellbeing, and between intelligence and wellbeing

		Causal effect estimates		Heterogeneity statistics		
	N SNPs	β (95% CI)	P	Q	df	P
**Years of schooling on wellbeing**						
IVW	54	0.057 (0.042, 0.074)	5.18E-13	336.7	53	6.83E-43
MR-Egger	54	-0.071 (-0.323, 0.180)	5.81E-01	330.1	52	4.38E-42
MR-Egger intercept	54	0.002 (-0.002, 0.006)	0.313	-	-	-
MR-Egger (SIMEX) [a]	54	-0.085 (-0.424, 0.252)	0.621	-	-	-
Weighted median	54	0.050 (0.023, 0.077)	2.29E-04	-	-	-
Weighted mode	54	0.067 (0.016, 0.119)	1.26E-02	-	-	-
**Wellbeing on years of schooling**						
IVW	147	0.206 (0.071, 0.341)	2.71E-03	574.9	146	3.19E-52
MR Egger estimate	147	0.049 (-0.651, 0.749)	0.890	574.1	145	2.15E-52
MR Egger intercept	147	0.001 (-0.004, 0.004)	0.655	-	-	-
MR-Egger (SIMEX) [a]	147	0.528 (-0.265, 1.32)	0.19	-	-	-
Weighted median	147	0.187 (0.063, 0.311)	2.80E-04	-	-	-
Weighted mode	147	0.236 (-0.094, 0.566)	0.163	-	-	-
**Intelligence on wellbeing**						
IVW	126	-0.004 (-0.028, 0.017)	0.713	688.5	125	7.58E-108
MR-Egger	126	0.003 (-0.096, 0.103)	0.946	688.4	124	5.54E-108
MR-Egger intercept	126	-0.001 (-0.003, 0.001)	0.883	-	-	-
MR-Egger (SIMEX) [a]	126	0.016 (-0.066, 0.098)	0.702	-	-	-
Weighted median	126	-0.001 (-0.018, 0.015)	0.876	-	-	-
Weighted mode	126	0.004 (-0.034, 0.041)	0.844	-	-	-
**Wellbeing on intelligence**						
IVW	128	0.199 (0.014, 0.390)	3.48E-02	665.4	127	6.72E-74
MR Egger estimate	128	-0.104 (-1.17, 0.960)	8.47E-01	663.7	126	5.81E-74
MR Egger intercept	128	0.001 (-0.006, 0.006)	0.571			
MR-Egger (SIMEX) [a]	128	0.688 (-0.375, 1.75)	0.207	-	-	-
Weighted median	128	0.301 (0.159, 0.443)	3.40E-05	-	-	-
Weighted mode	128	0.347 (-0.007, 0.702)	5.17E-02	-	-	-

Note: IVW= Inverse variance weighted estimate. Analyses conducted using the educational attainment discovery cohort (n=293,723). [a] Unweighted simulation extrapolation (SIMEX) correction applied

Supplementary Table 3: Univariable MR analyses assessing associations between wellbeing and educational attainment, and between wellbeing and intelligence following Steiger filtering

		Causal effect estimates		Heterogeneity statistics		
	N SNPs	β (95% CI)	P	Q	df	P
**Wellbeing on years of schooling**						
IVW	143	0.162 (0.033, 0.291)	0.014	499.6	142	1.34E-41
MR Egger estimate	143	0.065 (-0.605, 0.734)	0.850	499.9	141	2.28E-41
MR Egger intercept	143	0.000 (-0.005, 0.005)	0.998	-	-	-
Weighted median	143	0.177 (0.056, 0.299)	0.004	-	-	-
Weighted mode	143	0.236 (-0.107, 0.580)	0.180	-	-	-
**Wellbeing on intelligence**						
IVW	117	0.242 (0.098, 0.386)	9.66E-04	334.8	116	6.35E-23
MR Egger estimate	117	-0.163 (-0.976, 0.649)	6.95E-01	331.9	115	4.23E-23
MR Egger intercept	117	0.003 (-0.002, 0.008)	0.322			
Weighted median	117	0.297 (0.153, 0.441)	5.47E-05	-	-	-
Weighted mode	117	0.318 (-0.059, 0.696)	1.01E-01	-	-	-

Note: IVW= Inverse variance weighted estimate. Analyses conducted using the educational attainment discovery cohort (n=293,723).

Supplementary Table 4: F statistic and regression dilution I^2 statistic for the heterogeneity of SNP-exposure effects

**Exposure**	F	I^2 (unweighted)	I^2 (weighted)
Educational attainment on intelligence	38.44	0.38	0.52
Intelligence on educational attainment	42.72	0.48	0.51
Educational attainment on wellbeing	38.88	0.11	0
Intelligence on wellbeing	43.35	0.45	0.26
Wellbeing on educational attainment	40.78	0.35	0.35
Wellbeing on intelligence	40.83	0.35	0

Note: All based on analyses using educational attainment discovery GWAS (n=293,723).

Supplementary Table 5: Univariable MR analyses using intelligence GWAS without UKBiobank samples

		Causal effect estimates		Heterogeneity statistics		
	N SNPs	β (95% CI)	P	Q	df	P
**Years of schooling on intelligence**						
IVW	59	0.733 (0.624, 0.843)	2.40E-39	272.2	61	3.95E-28
MR-Egger	59	1.180 (0.597, 1.768)	2.13E-04	256.3	61	8.90E-26
MR-Egger intercept	63	-0.008 (-0.018, 0.002)	0.132	-	-	-
**Intelligence on years of schooling**						
IVW	98	0.517 (0.476, 0.558)	4.06E-134	1279.9	97	1.40E-200
MR-Egger estimate	98	-2.033 (-2.608, -1.459)	4.74E-10	714.9	96	2.56E-95
MR-Egger intercept	98	0.080 (0.078, 0.082)	8.74E-14	-	-	-
Weighted median	98	0.389 (0.351, 0.428)	9.43E-89	-	-	-
Weighted mode	98	0.259 (0.210, 0.309)	3.50E-17	-	-	-
**Intelligence on wellbeing**						
IVW	98	-0.019 (-0.032, -0.007)	0.003	418.8	97	1.02E-41
MR-Egger estimate	98	0.039 (-0.199, 0.277)	0.751	417.8	96	1.47E-41
MR-Egger intercept	98	-0.001 (-0.008, 0.006)	0.633	-	-	-
Weighted median	98	0.004 (-0.006, 0.015)	0.410	-	-	-
Weighted mode	98	0.007 (-0.011, 0.025)	0.451	-	-	-

Note: IVW= Inverse variance weighted estimate. UKBiobank samples not included in intelligence GWAS to remove samples that condition on socioeconomic status

Supplementary Table 6: Univariable MR analyses assessing impact of educational attainment and intelligence on positive affect

		Causal effect estimates		Heterogeneity statistics		
**Total effects**	N SNPs	β (95% CI)	P	Q	df	P
**Years of schooling on positive affect**						
Inverse variance weighted	54	0.037 (0.013, 0.061)	0.002	193.6	53	6.90E-18
MR-Egger	54	-0.044 (-0.335, 0.247)	0.766	192.4	52	5.42E-18
MR-Egger intercept	54	0.001 (-0.004, 0.006)	0.597	-	-	-
MR-Egger (SIMEX) [a]	54	-0.130 (-0.561, 0.302)	0.558	-	-	-
Weighted median	54	0.058 (0.020, 0.097)	0.003	-	-	-
Weighted mode	54	0.063 (-0.011, 0.137)	0.101	-	-	-
**Positive affect on years of schooling**						
Inverse variance weighted estimate	94	0.039 (-0.022, 0.100)	0.217	368.8	93	1.65E-34
MR Egger estimate	94	-0.257 (-0.787, 0.273)	0.343	363.8	92	5.43E-34
MR Egger intercept	94	0.003 (-0.004, 0.004)	0.263	-	-	-
MR-Egger (SIMEX) [a]	94	-0.254 (-0.985, 0.478)	0.498	-	-	-
Weighted median	94	0.000 (-0.118, 0.118)	1.00	-	-	-
Weighted mode	94	-0.120 (-0.458, 0.219)	0.490	-	-	-
**Intelligence on positive affect**						
Inverse variance weighted	126	-0.003 (-0.016, 0.010)	0.677	561.9	125	1.17E-56
MR-Egger	126	-0.017 (-0.153, 0.119)	0.808	561.8	124	5.93E-57
MR-Egger intercept	126	0.000 (-0.002, 0.002)	0.835	-	-	-
MR-Egger (SIMEX) [a]	126	-0.130 (-0.561, 0.302)	0.559	-	-	-
Weighted median	126	-0.001 (-0.023, 0.022)	0.927	-	-	-
Weighted mode	126	0.005 (-0.042, 0.051)	0.842	-	-	-
**Positive affect on intelligence**						
Inverse variance weighted estimate	83	0.953 (0.022, 0.168)	0.011	525.3	82	7.28E-66
MR Egger estimate	83	-0.695 (-1.48, 0.088)	0.086	499.8	81	1.39E-61
MR Egger intercept	83	0.007 (0.001, 0.01)	0.045	-	-	-
MR-Egger (SIMEX) [a]	83	-0.980 (-1.98, 0.02)	0.058	-	-	-
Weighted median	83	0.183 (0.034, 0.332)	0.016	-	-	-
Weighted mode	83	0.242 (-0.072, 0.556)	0.135	-	-	-

Note: Analyses conducted using the educational attainment discovery cohort (n=293,723).
[a] Weighted simulation extrapolation (SIMEX) correction. [b] Unweighted simulation extrapolation (SIMEX) correction

Supplementary Table 7: Univariable MR analyses assessing impact of educational attainment and intelligence on life satisfaction

		Causal effect estimates		Heterogeneity statistics		
**Total effects**	N SNPs	β (95% CI)	P	Q	df	P
**Years of schooling on life satisfaction**						
Inverse variance weighted	54	0.056 (0.028, 0.083)	6.29E-05	169.6	53	3.90E-14
MR-Egger	54	-0.020 (-0.330, 0.291)	9.01E-01	168.9	52	2.49E-14
MR-Egger intercept	54	0.001 (-0.004, 0.006)	0.631	-	-	-
Weighted median	54	0.067 (0.024, 0.111)	2.41E-03	-	-	-
Weighted mode	54	0.086 (0.004, 0.169)	4.54E-02	-	-	-
**Life satisfaction on years of schooling**						
Inverse variance weighted estimate	75	0.190 (0.123, 0.256)	2.06E-08	270.4	74	3.56E-24
MR Egger estimate	75	0.589 (-0.059, 1.24)	7.93E-02	264.9	73	1.38E-23
MR Egger intercept	75	-0.003 (-0.009, 0.009)	0.223	-	-	-
MR-Egger (SIMEX) [a]	75	0.776 (-0.049, 1.60)	0.069	-	-	-
Weighted median	75	0.181 (0.063, 0.299)	2.68E-03	-	-	-
Weighted mode	75	0.195 (-0.088, 0.478)	1.81E-02	-	-	-
**Intelligence on life satisfaction**						
Inverse variance weighted	126	0.003 (-0.012, 0.018)	0.687	449.9	125	3.11E-38
MR-Egger	126	0.015 (-0.126, 0.157)	0.831	449.8	124	1.69E-38
MR-Egger intercept	126	0.000 (-0.002, 0.002)	0.861	-	-	-
Weighted median	126	-0.009 (-0.036, 0.18)	0.510	-	-	-
Weighted mode	126	0.003 (-0.053, 0.060)	0.909	-	-	-
**Life satisfaction on intelligence**						
Inverse variance weighted estimate	63	0.222 (0.140, 0.304)	9.98E-08	332.1	62	1.41E-38
MR Egger estimate	63	-0.423 (-1.44, 0.601)	4.21E-01	323.7	61	1.87E-38
MR Egger intercept	63	0.006 (-0.002, 0.012)	0.214	-	-	-
MR-Egger (SIMEX) [a]	63	-0.284 (-1.36, 0.793)	0.607	-	-	-
Weighted median	63	0.239 (0.083, 0.395)	2.59E-03	-	-	-
Weighted mode	63	0.293 (-0.026, 0.612)	7.71E-02	-	-	-

Note: Analyses conducted using the educational attainment discovery cohort (n=293,723).
[a] Weighted simulation extrapolation (SIMEX) correction

Supplementary Table 8: Univariable MR analyses assessing impact of educational attainment and intelligence on depression

		Causal effect estimates		Heterogeneity statistics		
**Total effects**	N SNPs	β (95% CI)	P	Q	df	P
**Years of schooling on depression**						
Inverse variance weighted	54	-0.049 (-0.068, -0.032)	2.72E-08	252.2	53	1.03E-27
MR-Egger	54	0.086 (-0.154, 0.326)	4.84E-01	246.2	52	5.13E-27
MR-Egger intercept	54	-0.002 (-0.006, 0.002)	0.265	-	-	-
MR-Egger (SIMEX) [a]	54	0.086 (-0.265, 0.437)	0.633	-	-	-
Weighted median	54	-0.047 (-0.077, -0.018)	1.39E-03	-	-	-
Weighted mode	54	-0.019 (-0.071, 0.032)	4.63E-01	-	-	-
**Depression on years of schooling**						
Inverse variance weighted estimate	114	-0.172 (-0.241, -0.102)	1.45E-06	456.2	113	8.30E-43
MR Egger estimate	114	-0.176 (-0.871, (0.520)	6.21E-01	456.4	112	4.09E-43
MR Egger intercept	114	0.000 (-0.006, 0.006)	0.223	-	-	-
MR-Egger (SIMEX) [a]	114	-0.661 (-1.55, 0.230)	0.149	-	-	-
Weighted median	114	-0.181 (-0.314, -0.049)	7.28E-03	-	-	-
Weighted mode	114	-0.261 (-0.609, 0.086)	1.43E-01	-	-	-
**Intelligence on depression**						
Inverse variance weighted	126	0.001 (-0.004, 0.015)	0.279	632.1	125	8.97E-69
MR-Egger	126	0.014 (-0.093, 0.121)	0.799	632.1	124	4.17E-69
MR-Egger intercept	126	0.000 (-0.002, 0.002)	0.873	-	-	-
MR-Egger (SIMEX) [a]	126	0.020 (-0.121, 0.161)	0.785	-	-	-
Weighted median	126	0.007 (-0.011, 0.024)	0.451	-	-	-
Weighted mode	126	0.001 (-0.036, 0.039)	0.943	-	-	-
**Depression on intelligence**						
Inverse variance weighted estimate	98	-0.222 (-0.304, -0.140)	9.98E-08	332.1	62	1.41E-38
MR Egger estimate	98	0.423 (-0.601, 1.44)	4.21E-01	323.7	61	1.87E-38
MR Egger intercept	98	-0.006 (-0.012, 0.002)	0.214	-	-	-
MR-Egger (SIMEX) [b]	98	0.788 (-0.491, 2.07)	0.230	-	-	-
Weighted median	98	-0.239 (-0.395, -0.083)	2.59E-03	-	-	-
Weighted mode	98	-0.293 (-0.612, 0.026)	7.71E-02	-	-	-

Note: Analyses conducted using the educational attainment discovery cohort (n=293,723).
[a] Weighted simulation extrapolation (SIMEX) correction. [b] Weighted simulation extrapolation (SIMEX) correction

Supplementary Table 9: Univariable MR analyses assessing impact of educational attainment and intelligence on neuroticism

		Causal effect estimates		Heterogeneity statistics		
**Total effects**	N SNPs	β (95% CI)	P	Q	df	P
**Years of schooling on neuroticism**						
Inverse variance weighted	54	-0.099 (-0.127, -0.072)	1.56E-12	360.9	53	2.11E-47
MR-Egger	54	0.027 (-0.433, 0.487)	9.07E-01	358.9	52	1.93E-47
MR-Egger intercept	54	-0.002 (-0.010, 0.006)	0.587	-	-	-
MR-Egger (SIMEX) [a]	54	-0.089 (-0.704, 0.526)	0.777	-	-	-
Weighted median	54	-0.084 (-0.133, -0.035)	6.99E-04	-	-	-
Weighted mode	54	-0.070 (-0.154, 0.014)	1.07E-01	-	-	-
**Neuroticism on years of schooling**						
Inverse variance weighted estimate	113	-0.126 (-0.171, -0.082)	3.07E-08	391.3	112	1.26E-32
MR Egger estimate	113	-0.212 (-0.575, 0.151)	2.55E-01	390.5	111	8.91E-33
MR Egger intercept	113	0.001 (-0.005, 0.005)	0.636	-	-	-
MR-Egger (SIMEX) [a]	113	-0.568 (-1.06, -0.077)	0.025	-	-	-
Weighted median	113	-0.111 (-0.193, -0.030)	7.37E-03	-	-	-
Weighted mode	113	0.078 (-0.146, 0.300)	5.01E-01	-	-	-
**Intelligence on neuroticism**						
Inverse variance weighted	126	-0.015 (-0.030, 0.003)	0.055	632.1	125	8.97E-69
MR-Egger	126	-0.041 (-0.208, 0.125)	0.627	632.1	124	4.17E-69
MR-Egger intercept	126	0.000 (-0.003, 0.003)	0.751	-	-	-
MR-Egger (SIMEX) [a]	126	-0.052 (-0.188, 0.085)	0.461	-	-	-
Weighted median	126	0.000 (-0.028, 0.029)	0.978	-	-	-
Weighted mode	126	0.004 (-0.057, 0.065)	0.909	-	-	-
**Neuroticism on intelligence**						
Inverse variance weighted estimate	96	-0.180 (-0.234, -0.126)	6.31E-11	332.1	62	1.41E-38
MR Egger estimate	96	-0.058 (-0.618, 0.503)	8.40E-01	323.7	61	1.87E-38
MR Egger intercept	96	-0.001 (-0.007, 0.005)	0.661	-	-	-
MR-Egger (SIMEX) [b]	96	-0.164 (-0.807, 0.480)	0.619	-	-	-
Weighted median	96	-0.186 (-0.291, -0.082)	4.85E-04	-	-	-
Weighted mode	96	-0.152 (-0.431, 0.126)	2.84E-01	-	-	-

Note: Analyses conducted using the educational attainment discovery cohort (n=293,723).
[a] Weighted simulation extrapolation (SIMEX) correction. [b] Weighted simulation extrapolation (SIMEX) correction

Supplementary Table 10: Comparison of main variable scores between ALSPAC subsamples.

	N	Sex	Subjective Happiness	Life Satisfaction	University degree	Intelligence
**Subsample**		% Female	Mean, SD	Mean, SD	% With degree	Mean, SD
Wellbeing only[a]	4,254	65.9	4.88 (1.29)	24.05 (6.98)	-	-
Education and wellbeing[b]	3,788	66.1	4.89 (1.28)	24.17 (6.96)	64.0	-
Intelligence and wellbeing[c]	3,179	64.3	4.90 (1.27)	24.33 (6.89)	-	107.68 (16.03)
Complete data[d]	2,844	64.6	4.92 (1.27)	24.37 (6.84)	66.6	107.82 (16.05)

Note: [a]Data on subjective happiness and life satisfaction at 26 years. [b]Data on educational attainment, subjective happiness, and life satisfaction at 26 years. [c]Data on intelligence at 8 years, and subjective happiness and life satisfaction at 26 years. [d]Data on educational attainment at 26 years, intelligence at 8 years, and subjective happiness and life satisfaction at 26 years.

Supplementary Table 11: Description of study variables in ALSPAC

Construct	Scale used	Number of items	Scoring	Composite creation / notes	Completed by	Age
**Predictors**						
Educational attainment	1 item, "Do you have a university degree?"	1	0="No", 1="Yes", 2="Still at university"	Those responding 2 excluded*	Participant	26
Intelligence	Wechsler Intelligence Scale for Children (WISC-III)		Sum across various verbal and performance tests	Higher score equates to higher IQ	Participant	8
Family income	Reported family income per week	1	Less than £100, £100 - £199, £200-£299, £300 - £399, £400 or more, or don't know	Continuous score	Mother	2 years 9 months
**Outcomes**						
Subjective happiness	Subjective Happiness Scale	4	1-7 ("Not a very happy person" – "A very happy person)	Reverse code final item and then take the mean. Higher score reflects greater happiness	Participant	23
Life Satisfaction	Satisfaction with Life Scale	5	1-7 ("Strongly disagree" - "Strongly agree")	Sum all items. Higher score represents higher life satisfaction	Participant	23

Note: Those who responded 'still at university' (*n*=200) were excluded because such individuals at 26 years would not necessarily represent those who followed the typical educational trajectory. For example, individuals may have taken a break from education and returned, or re-taking courses. Including such individuals may therefore have skewed analyses or created noise between the observational and MR findings.

Supplementary Table 12: Observational regression results assessing linear associations between educational attainment and wellbeing, and between intelligence and wellbeing (unstandardised)

	Unadjusted		Adjusted using IPW		Adjusted using multiple imputation (*n*=4,298)		Complete cases (*n*=2,844)	
	β (95% CI)	P	β (95% CI)	P	β (95% CI)	P	β (95% CI)	P
**Subjective happiness**								
Model 1								
University degree	0.004 (-0.080, 0.090)	0.920	-0.028 (-0.126, 0.070)	0.581	-0.026 (-0.131, 0.079)	0.624	-0.021 (-0.101, 0.060)	0.615
Model 2								
Intelligence	-0.003 (-0.006, -0.000)	0.038	-0.003 (-0.006, -0.000)	0.033	-0.004 (-0.006, -0.002)	4.58E-06	-0.003 (-0.006, -0.000)	0.008
Model 3								
University degree	-0.291 (-0.436, -0.145)	9.18E-05	-0.282 (-0.447, -0.117)	7.98E-04	-0.281 (-0.454, -0.109)	0.001	-0.313 (-0.450, -0.175)	8.54E-06
Sex	-0.239 (-0.382, -0.096)	0.001	-0.180 (-0.347, -0.012)	0.036	-0.123 (-0.274, 0.028)	0.111	-0.236 (-0.372, -0.100)	6.63E-04
University degree*Sex	0.447 (0.268, 0.626)	1.05E-06	0.395 (0.189, 0.601)	1.69E-04	0.397 (0.179, 0.615)	3.60E-04	0.443 (0.274, 0.612)	3.08E-07
Model 4								
Intelligence	-0.007 (-0.011, -0.002)	0.003	-0.005 (-0.010, -0.000)	0.027	-0.004 (-0.006, -0.001)	0.003	-0.007 (-0.011, -0.003)	4.27E-04
Sex	-0.612 (-1.23, 0.008)	0.053	-0.325 (0.979, 0.328)	0.329	-0.176 (-0.204, 0.557)	0.364	-0.634 (-1.18, -0.093)	0.022
Intelligence*Sex	0.006 (0.000, 0.012)	0.029	0.004 (-0.002, 0.010)	0.223	0.000 (-0.004, 0.003)	0.670	0.006 (0.001, 0.011)	0.013
**Life satisfaction**								
Model 5								
University degree	0.031 (0.017, 0.046)	3.66E-05	0.029 (0.014, 0.045)	2.40E-04	0.029 (0.019, 0.039)	2.66E-08	0.030 (0.017, 0.043)	5.27E-06

Model 6								
Intelligence	0.031 (0.017, 0.046)	3.66E-05	0.029 (0.014, 0.045)	2.40E-04	0.029 (0.019, 0.039)	2.66E-08	0.030 (0.017, 0.043)	5.27E-06
Model 7								
University degree	0.529 (-0.254, 1.31)	0.185	0.492 (-0.394, 1.38)	0.276	0.104 (-0.828, 1.04)	0.826	0.636 (-0.105, 1.38)	0.092
Sex	-0.147 (-0.917, 0.623)	0.701	-0.056 (0.956, 0.844)	0.903	-0.238 (-1.06, 0.579)	0.568	-0.055 (-0.787, 0.677)	0.883
University degree*Sex	1.74 (0.776, 2.71)	4.07E-04	1.64 (0.538, 2.75)	0.004	1.96 (0.782, 3.13)	0.001	1.49 (0.580, 2.40)	0.001
Model 8								
Intelligence	0.012 (-0.012, 0.035)	0.340	0.017 (-0.008, 0.043)	0.174	0.029 (0.015, 0.043)	4.21E-05	0.014 (-0.008, 0.035)	0.206
Sex	-2.92 (-6.27, 0.424)	0.087	-1.39 (-4.91, 2.13)	0.439	-1.27 (-0.805, 3.35)	0.230	-2.12 (-5.04, 0.800)	0.155
Intelligence*Sex	0.036 (0.006, 0.067)	0.019	0.023 (-0.010, 0.055)	0.158	0.001 (-0.021, 0.019)	0.923	0.029 (0.002, 0.056)	0.035

Note:[†]FDR. IPW= Inverse probability weighting. Sex coded as 0=Male and 1=Female, analyses therefore used male as the reference. In unadjusted models and models adjusted for IPW, n=3,788 for educational attainment and n=3,179 for intelligence.

Supplementary Table 13: Observational regression results assessing linear associations between educational attainment and wellbeing, and between intelligence and wellbeing with adjustments for family income

	Unadjusted (unstandardised)		Adjusted for family income (unstandardised)		Unadjusted (standardised)		Adjusted for family income (standardised)	
	β (95% CI)	P	β (95% CI)	P	β (95% CI)	P	β (95% CI)	P
**Subjective happiness**								
Model 1								
University degree	-0.009 (-0.108, 0.089)	0.847	-0.073 (-0.176, 0.030)	0.163	-0.007 (-0.084, 0.069)	0.847	-0.049 (-0.130, 0.030)	0.222
Family income	-	-	0.071 (0.030, 0.112)	7.57E-05	-	-	0.072 (0.031, 0.112)	5.26E-04
Model 2								
Intelligence	-0.004 (-0.007, -0.001)	0.022	-0.005 (-0.008, -0.002)	0.002	-0.002 (-0.005, -0.001)	0.022	-0.004 (-0.006, -0.001)	0.002
Family income	-	-	0.075 (0.030, 0.119)	8.70E-03	-	-	0.073 (0.030, 0.117)	8.70E-03
Model 3								
University degree	-0.270 (-0.435, -0.105)	0.001	-0.320 (-0.487, -0.152)	1.83E-05	-0.210 (-0.338, -0.081)	0.001	-0.248 (-0.378, -0.118)	1.83E-03
Sex	-0.204 (-0.370, -0.039)	0.015	-0.192 (-0.357, -0.027)	0.022	-0.159 (-0.287, -0.030)	0.015	-0.149 (-0.278, -0.021)	0.022
University degree*Sex	0.402 (0.197, 0.608)	1.29E-05	0.395 (0.190, 0.600)	1.65E-04	0.312 (0.153, 0.472)	1.29E-05	0.307 (0.147, 0.466)	1.65E-04
Family income	-	-	0.072 (0.03, 0.114)	5.60E-04	-	-	0.071 (0.031, 0.111)	5.60E-04
Model 4								
Intelligence	-0.007 (-0.011, -0.002)	0.003	-0.008 (-0.013, -0.003)	0.001	-0.006 (-0.010, -0.002)	0.001	-0.007 (-0.011, -0.003)	1.86E-04
Sex	-0.612 (-1.23, 0.008)	0.053	-0.769 (-1.460, -0.078)	0.029	-0.597 (-1.133, -0.061)	0.029	-0.586 (-1.122, -0.051)	0.032
Intelligence*Sex	0.006 (0.000, 0.012)	0.029	0.008 (0.001, 0.014)	0.017	0.005 (0.000, 0.001)	0.017	0.006 (0.001, 0.011)	0.018

Family income	-	-	0.075 (0.031, 0.119)	8.56E-04	-	-	0.073 (0.030, 0.117)	8.56E-04
**Life satisfaction**								
Model 5								
University degree	1.718 (1.189, 2.247)	2.11E-10	1.158 (0.609, 1.705)	3.58E-05	0.246 (0.170, 0.321)	2.11E-10	0.168 (0.089, 0.247)	2.95E-05
Family income	-	-	0.726 (0.506, 0.946)	1.12E-10	-	-	0.132 (0.092, 0.172)	8.73E-11
Model 6								
Intelligence	0.032 (0.016, 0.490)	1.20E-03	0.017 (0.000, 0.034)	0.049	0.005 (0.002, 0.007)	1.20E-03	0.002 (0.000, 0.005)	0.049
Family income	-	-	0.811 (0.058, 1.048)	1.92E-11	-	-	0.146 (0.138, 0.189)	1.92E-11
Model 7								
University degree	0.675 (-0.208, 1.559)	0.134	0.166 (-0.724, 1.055)	0.715	0.010 (-0.030, 0.223)	0.134	0.024 (-0.104, 0.151)	0.715
Sex	0.084 (-0.080, 0.970)	0.851	0.207 (-0.672, 1.086)	0.644	0.012 (-0.15, 0.139)	0.851	0.030 (-0.096, 0.156)	0.644
University degree*Sex	1.592 (0.491, 2.692)	0.005	1.518 (0.426, 2.610)	0.006	0.228 (0.070, 0.386)	0.005	0.217 (0.061, 0.374)	0.006
Family income	-	-	0.747 (0.527, 0.967)	3.11E-11	-	-	0.135 (0.095, 0.174)	3.11E-11
Model 8								
Intelligence	0.011 (-0.016, 0.037)	0.426	0.008 (-0.013, 0.003)	0.678	0.002 (-0.002, 0.005)	0.426	-0.005 (-0.004, 0.003)	0.760
Sex	-3.225 (-6.929, 0.480)	0.088	-3.071 (-0.030, 0.022)	0.101	-0.462 (-0.993, 0.069)	0.089	-0.440 (-0.966, 0.009)	0.101

Intelligence*Sex	0.040 (0.006, 0.073)	0.022	0.038 (0.005, 0.072)	0.024	0.006 (0.001, 0.011)	0.022	0.006 (0.000, 0.010)	0.024
Family income	-	-	0.818 (0.583, 1.053)	1.15E-11	-	-	0.117 (0.094, 0.151)	1.15E-11

Note: Sex coded as 0=Male and 1=Female, analyses therefore used male as the reference. In unadjusted models and models adjusted for IPW, n=3,788 for educational attainment and n=3,179 for intelligence.

Supplementary Table 14: Observational regression results from linear, quadratic, cubic and quartic models assessing associations between intelligence and wellbeing

	Linear model			Quadratic model			Cubic model			Quartic model		
	β	SE	P	β	SE	P	β	SE	P	β	SE	P
**Subjective happiness**												
Intercept (β0)	0.262	0.119	0.028	0.018	0.018	0.295	0.018	0.018	0.295	0.018	0.018	0.295
Intelligence (linear)	-0.002	0.001	0.038	-2.05	0.987	0.038	-2.05	0.987	0.038	-2.05	0.987	0.038
$Intelligence^2$ (quadratic)	-	-	-	-1.09	0.987	0.271	-1.09	0.987	0.271	-1.09	0.987	0.271
$Intelligence^3$ (Cubic)	-	-	-	-	-	-	-.238	0.987	0.809	-0.238	0.987	0.809
$Intelligence^4$ (Quartic)	-	-	-	-	-	-	-	-	-	-1.35	0.987	0.172
Adjusted R^2	0.001			0.001			0.001			0.001		
RMSE	0.987			0.986			0.986			0.986		
AIC	8941.877			8942.666			8944.607			8944.740		
BIC	8960.070			8966.923			8974.929			8981.126		
**Life satisfaction**												
Intercept	-0.460	0.119	0.001	0.025	0.018	0.149	0.025	0.018	0.149	0.025	0.018	0.149
Intelligence (linear)	0.005	0.001	3.66E-05	4.07	0.984	3.67E-05	4.07	0.984	3.67E-05	4.07	0.984	3.67E-05
$Intelligence^2$ (quadratic)	-	-	-	0.237	0.984	0.809	0.237	0.984	0.809	0.237	0.984	0.809
$Intelligence^3$ (Cubic)	-	-	-	-	-	-	0.769	0.984	0.435	0.769	0.984	0.435
$Intelligence^4$ (Quartic)	-	-	-	-	-	-	-	-	-	-1.59	0.984	0.106

Adjusted R^2	0.005	0.005	0.005	0.005
RSME	0.984	0.984	0.984	0.984
AIC	8925.193	8927.135	8928.524	8927.904
BIC	8943.386	8951.392	89.58.846	8964.290

RMSE = Residual standard error; AIC = Akaike information criterion; BIC = Bayesian information criterion.

Supplementary Table 15: Univariable MR analyses assessing bidirectional associations between educational attainment (using discovery and replication cohort, n=405,072) and intelligence

		Causal effect estimates		Heterogeneity statistics		
	N SNPs	β (95% CI)	P	Q	df	P
**Years of schooling on intelligence**						
Inverse variance weighted estimate	122	0.775 (0.710, 0.842)	4.09E-117	443.2	121	<0.001
MR Egger estimate	122	1.06 (0.784, 1.33)	7.01E-12	427.7	120	<0.001
MR Egger intercept	122	-0.004 (-0.009, -0.002)	0.042	-	-	-
MR-Egger (SIMEX) [a]	122	1.36 (1.04, 1.68)	5.98E-14	-	-	-
Weighted median	122	0.682 (0.614, 0.751)	4.56E-84	-	-	-
Weighted mode	122	0.668 (0.493, 0.844)	1.45E-11	-	-	-
**Intelligence on years of schooling**						
Inverse variance weighted estimate	144	0.436 (0.399, 0.472)	1.33E-119	813.5	143	<0.001
MR Egger estimate	144	0.510 (0.341, 0.679)	2.47E-08	809.1	142	<0.001
MR Egger intercept	144	-0.003 (-0.006, 0.001)	0.150	-	-	-
MR-Egger (SIMEX) [a]	144	0.790 (0.552, 1.03)	9.93E-10	-	-	-
Weighted median	144	0.339 (0.306, 0.371)	2.15E-93	-	-	-
Weighted mode	144	0.296 (0.229, 0.364)	1.49E-14	-	-	-

Note: Analyses conducted using the educational attainment discovery and replication cohort (n=405,072).

[a] Weighted simulation extrapolation (SIMEX) correction applied

Supplementary Table 16: Results from MRLap tests of impact of sample overlap

		Univariable MR Estimate		MR Lap Corrected Estimate		Test of difference	
	N SNPs	Beta (95% CI)	p-value	Beta (95% CI)	p-value	t_{diff}	p-value
Education (p<5x10-6, exc. 23andMe) -> Wellbeing	271	0.039 (0.026, 0.051)	<0.001	0.046 (0.030, 0.063)	<0.001	-3.33	<0.001
Intelligence -> Wellbeing	136	-0.003 (-0.023, 0.018)	0.81	-0.005 (-0.031, 0.022)	0.73	0.68	0.49

Supplementary Table 17: List of SNPs used for multivariable MR

			Association with years of schooling			Association with intelligence			Association with wellbeing		
SNP	A1	A2	BETA	SE	P	BETA	SE	P	BETA	SE	P
rs10006235	C	T	0.010	0.002	1.45E-07	0.003	0.003	0.304	0.001	0.001	0.122
rs1008078	T	C	-0.017	0.002	1.20E-23	-0.011	0.003	1.11E-04	-0.001	0.001	0.167
rs10189857	G	A	-0.017	0.002	6.70E-24	-0.019	0.003	4.91E-12	0.000	0.001	0.458
rs10189912	A	G	-0.016	0.002	6.49E-20	-0.019	0.003	1.22E-11	-0.003	0.001	4.90E-04
rs1035578	A	G	-0.011	0.002	1.33E-10	-0.009	0.003	0.002	-0.001	0.001	0.241
rs10483349	G	A	0.019	0.002	1.54E-17	0.013	0.004	3.28E-04	0.000	0.001	0.489
rs10772644	G	C	-0.016	0.003	1.50E-09	-0.008	0.004	0.057	0.000	0.001	0.471
rs10779271	A	G	0.004	0.002	0.015	0.016	0.003	2.17E-08	0.000	0.001	0.359
rs10831912	C	T	0.011	0.002	5.19E-11	0.009	0.003	0.022	0.000	0.001	0.308
rs10917152	C	T	-0.006	0.003	0.0128	-0.024	0.004	2.23E-09	0.001	0.001	0.345
rs10954779	C	T	0.010	0.002	1.06E-08	0.016	0.003	3.04E-09	0.001	0.001	0.091
rs1106761	A	G	-0.017	0.002	7.71E-22	-0.018	0.003	9.12E-10	0.002	0.001	0.010
rs11076962	T	C	0.007	0.002	2.00E-04	0.017	0.003	2.57E-08	0.004	0.001	2.01E-04
rs11191193	G	A	-0.018	0.002	5.51E-23	-0.023	0.003	1.29E-15	-0.001	0.001	0.083
rs112780312	G	A	0.007	0.002	1.37E-04	0.018	0.003	3.66E-09	-0.001	0.001	0.297
rs1145123	C	T	-0.004	0.002	0.020	-0.021	0.003	1.20E-13	0.000	0.001	0.484
rs115064	C	T	-0.006	0.002	4.95E-04	-0.016	0.003	1.07E-08	0.002	0.001	0.036
rs11605348	G	A	-0.004	0.002	0.020	0.017	0.003	9.73E-09	-0.006	0.001	7.81E-11

rs11634187	T	G	0.006	0.002	0.010	0.022	0.004	1.12E-08	0.002	0.001	0.044
rs11646221	T	G	0.011	0.002	3.01E-11	0.018	0.003	1.57E-10	-0.006	0.001	7.82E-12
rs11678106	C	T	-0.004	0.002	0.011	-0.016	0.003	4.62E-09	-0.001	0.001	0.091
rs11720523	C	A	-0.013	0.002	1.54E-14	-0.018	0.003	3.89E-11	-0.002	0.001	0.021
rs11898362	G	A	0.007	0.002	6.84E-05	0.018	0.003	2.25E-09	0.002	0.001	0.047
rs12190777	A	G	0.009	0.002	5.45E-06	0.017	0.003	3.63E-08	-0.002	0.001	0.025
rs12410444	G	A	0.019	0.002	4.86E-24	0.016	0.003	9.40E-08	0.000	0.001	0.374
rs12470949	C	T	0.003	0.002	0.16	0.017	0.003	1.32E-08	0.003	0.001	0.003
rs12514965	C	T	-0.015	0.002	5.35E-14	-0.014	0.003	1.18E-05	0.004	0.001	4.1E-04
rs12535854	G	C	0.005	0.002	0.003	0.018	0.003	6.73E-10	0.001	0.001	0.245
rs12761761	T	C	0.016	0.002	8.63E-15	0.018	0.003	2.44E-08	0.002	0.001	0.045
rs1280049	A	C	0.005	0.002	0.002	0.015	0.003	3.92E-08	0.001	0.001	0.172
rs12886584	C	T	-0.007	0.002	0.002	-0.021	0.004	9.41E-09	0.000	0.001	0.491
rs12962421	A	G	-0.009	0.002	1.16E-07	-0.003	0.003	0.285	0.001	0.001	0.074
rs12969294	G	A	0.018	0.002	1.13E-23	0.005	0.003	0.120	0.008	0.001	1.96E-15
rs13010288	G	T	-0.020	0.003	1.04E-14	-0.002	0.004	0.621	-0.001	0.001	0.255
rs13071190	T	C	0.007	0.002	3.83E-05	0.018	0.003	5.55E-10	-0.001	0.001	0.263
rs13212044	G	T	0.009	0.002	1.38E-05	0.018	0.003	1.46E-08	0.003	0.001	0.006
rs13223152	G	A	-0.006	0.002	2.24E-04	-0.018	0.003	2.34E-10	0.003	0.001	0.001
rs13253386	T	G	-0.005	0.002	0.005	-0.020	0.003	2.37E-13	-0.003	0.001	0.002
rs13276212	T	G	0.004	0.002	0.033	0.015	0.003	4.48E-08	0.001	0.001	0.282

rs13421974	C	T	-0.012	0.002	1.33E-11	-0.010	0.003	5.34E-04	-0.001	0.001	0.248
rs1362739	A	C	0.010	0.002	1.16E-08	0.021	0.003	1.83E-14	0.004	0.001	2.54E-05
rs1369429	T	C	0.001	0.002	0.477	0.018	0.003	1.15E-09	-0.004	0.001	1.54E-05
rs1378214	T	C	-0.013	0.002	1.90E-14	-0.002	0.003	0.4367	-0.004	0.001	5.54E-05
rs1408579	C	T	-0.007	0.002	1.12E-05	-0.016	0.003	5.23E-09	0.001	0.001	0.170
rs144246	A	G	0.009	0.002	8.79E-08	0.015	0.003	4.91E-08	-0.001	0.001	0.194
rs1589652	G	A	-0.002	0.002	0.183	-0.017	0.003	5.82E-10	-0.002	0.001	0.032
rs166820	G	A	-0.009	0.002	3.12E-05	-0.024	0.004	1.37E-11	-0.002	0.001	0.056
rs16845580	C	T	-0.014	0.002	1.54E-15	-0.017	0.003	1.27E-09	-0.002	0.001	0.008
rs17128425	T	A	-0.007	0.003	0.0169	-0.026	0.005	1.87E-08	0.002	0.002	0.074
rs17425572	G	A	-0.012	0.002	6.89E-13	-0.011	0.003	9.06E-05	-0.001	0.001	0.102
rs1831539	C	T	0.005	0.002	0.002	0.017	0.003	4.72E-10	-0.002	0.001	0.017
rs1840847	A	G	0.005	0.002	0.005	0.016	0.003	1.44E-08	-0.001	0.001	0.237
rs1906252	C	A	-0.022	0.002	6.88E-40	-0.032	0.003	7.48E-31	-0.001	0.001	0.095
rs1972860	G	A	0.010	0.002	2.05E-08	0.018	0.003	2.09E-09	-0.003	0.001	0.003
rs2007176	T	C	0.015	0.002	2.29E-18	0.015	0.003	2.64E-08	0.001	0.001	0.167
rs2008514	A	G	-0.017	0.002	7.43E-22	-0.029	0.003	1.25E-24	0.001	0.001	0.273
rs2071407	C	T	0.009	0.002	2.25E-07	0.022	0.003	1.52E-14	-0.006	0.001	8.27E-10
rs2072490	C	T	-0.004	0.002	0.016	-0.017	0.003	5.93E-10	0.000	0.001	0.318
rs2285640	G	A	-0.006	0.002	5.47E-04	-0.018	0.003	2.38E-10	0.004	0.001	2.49E-05
rs2309812	T	C	0.021	0.002	1.80E-32	0.023	0.003	9.95E-16	0.001	0.001	0.218

rs2373353	G	A	0.007	0.002	3.54E-05	0.016	0.003	1.56E-08	0.000	0.001	0.356
rs2393967	A	C	-0.011	0.002	1.61E-09	-0.019	0.003	2.70E-10	0.000	0.001	0.480
rs2450333	A	G	-0.012	0.002	1.61E-12	-0.019	0.003	1.73E-11	0.002	0.001	0.024
rs2456973	C	A	0.017	0.002	2.99E-22	0.015	0.003	4.31E-07	-0.001	0.001	0.292
rs2478286	G	C	0.009	0.002	9.65E-06	0.026	0.003	1.64E-16	0.000	0.001	0.446
rs2558096	T	G	-0.005	0.002	0.009	-0.016	0.003	1.74E-08	-0.002	0.001	0.013
rs2647995	C	T	0.007	0.002	1.71E-04	0.020	0.003	8.68E-11	-0.003	0.001	0.005
rs2678210	T	C	0.010	0.002	7.43E-08	0.019	0.003	6.97E-10	0.002	0.001	0.011
rs2726491	G	A	0.013	0.002	1.40E-14	0.028	0.003	4.17E-23	-0.004	0.001	1.63E-06
rs2836921	A	G	0.005	0.002	0.004	0.020	0.003	6.54E-12	-0.001	0.001	0.184
rs28620532	G	A	0.004	0.002	0.012	0.016	0.003	1.51E-08	-0.003	0.001	0.003
rs287879	G	A	0.006	0.002	0.001	0.019	0.003	8.47E-10	0.001	0.001	0.149
rs2920940	T	C	-0.007	0.002	7.51E-04	-0.025	0.003	2.76E-14	0.003	0.001	0.001
rs297578	A	G	0.007	0.002	1.34E-04	0.018	0.003	1.73E-09	0.001	0.001	0.120
rs3095075	A	G	-0.012	0.002	2.10E-12	-0.008	0.003	0.006	-0.003	0.001	0.001
rs31768	A	T	0.004	0.002	0.026	0.018	0.003	2.65E-09	0.002	0.001	0.012
rs329672	C	T	-0.007	0.002	2.80E-05	-0.017	0.003	1.00E-09	-0.003	0.001	2.54E-04
rs34316	C	A	-0.020	0.002	3.35E-30	-0.021	0.003	2.82E-14	0.004	0.001	9.69E-06
rs34344888	A	G	-0.014	0.002	6.20E-15	-0.006	0.003	0.043	-0.001	0.001	0.206
rs35731967	T	C	0.007	0.002	0.007	0.022	0.004	2.38E-09	0.001	0.001	0.194
rs36033	T	C	0.003	0.002	0.059	0.016	0.003	1.02E-08	0.001	0.001	0.231

rs4240470	G	C	-0.013	0.002	7.44E-13	-0.009	0.003	0.003	-0.002	0.001	0.052
rs4244613	A	G	-0.010	0.002	2.12E-08	-0.015	0.005	0.0042	-0.002	0.001	0.008
rs4463213	A	G	0.006	0.002	6.03E-04	0.019	0.003	3.00E-12	-0.005	0.001	4.47E-07
rs4468571	A	G	-0.013	0.002	5.55E-14	-0.006	0.003	0.0375	-0.005	0.001	5.30E-08
rs4478846	T	C	0.018	0.002	8.48E-16	0.013	0.004	0.005	-0.001	0.001	0.312
rs4484297	C	G	0.005	0.002	0.015	0.018	0.003	7.45E-09	0.000	0.001	0.349
rs4493682	C	G	0.011	0.002	1.70E-06	0.008	0.003	0.0165	0.002	0.001	0.078
rs4725065	A	G	-0.012	0.002	5.94E-12	-0.017	0.003	1.52E-09	0.001	0.001	0.080
rs4731392	A	G	-0.009	0.002	5.51E-07	-0.022	0.003	2.69E-13	0.000	0.001	0.344
rs4800490	C	A	0.014	0.002	3.04E-16	0.008	0.003	0.004	-0.001	0.001	0.240
rs4821995	A	G	0.005	0.002	0.007	0.016	0.003	2.62E-08	0.003	0.001	0.001
rs4852252	C	T	0.004	0.002	0.030	0.021	0.003	3.84E-14	-0.003	0.001	0.001
rs4863692	G	T	-0.014	0.002	3.77E-15	-0.015	0.003	4.81E-07	-0.002	0.001	0.039
rs4974424	A	G	-0.015	0.002	1.98E-10	-0.017	0.004	5.38E-06	0.000	0.001	0.462
rs4981713	T	G	0.009	0.002	1.15E-07	0.016	0.003	7.23E-09	0.005	0.001	5.01E-08
rs523934	A	G	0.015	0.002	1.23E-16	0.015	0.003	1.23E-07	0.000	0.001	0.479
rs538628	G	C	0.017	0.002	3.94E-15	0.015	0.003	2.03E-05	0.005	0.001	7.54E-07
rs55754731	C	T	-0.011	0.002	2.20E-06	-0.021	0.004	6.06E-09	-0.003	0.001	0.006
rs55763037	A	G	0.008	0.002	5.66E-05	0.018	0.003	3.74E-08	0.002	0.001	0.055
rs566237	G	A	0.003	0.002	0.076	0.019	0.003	1.82E-10	0.000	0.001	0.413
rs5750830	C	A	-0.008	0.002	6.44E-05	-0.023	0.003	2.46E-13	0.000	0.001	0.415

rs58694847	C	G	-0.014	0.002	1.37E-12	-0.010	0.003	0.001	-0.002	0.001	0.011
rs59142272	G	A	-0.011	0.002	1.33E-06	-0.023	0.004	7.32E-10	0.003	0.001	0.016
rs600806	A	G	-0.012	0.002	1.58E-09	-0.019	0.003	3.57E-10	0.002	0.001	0.033
rs6019535	G	A	-0.008	0.002	1.56E-05	-0.025	0.003	3.28E-17	0.006	0.001	8.51E-09
rs60262711	T	C	0.007	0.002	0.002	0.016	0.003	1.65E-08	0.001	0.001	0.244
rs61160187	A	G	-0.020	0.002	1.17E-30	-0.012	0.003	3.01E-05	-0.001	0.001	0.176
rs62181012	T	C	0.006	0.002	0.002	0.021	0.004	1.73E-09	-0.001	0.001	0.100
rs62263923	A	G	-0.015	0.002	6.59E-17	-0.008	0.003	0.004	0.000	0.001	0.431
rs6508220	G	A	0.011	0.002	9.97E-11	0.023	0.003	9.56E-17	0.005	0.001	1.49E-08
rs6535809	G	A	-0.009	0.002	1.17E-07	-0.020	0.003	6.65E-13	-0.001	0.001	0.085
rs6539284	T	C	-0.011	0.002	3.53E-10	-0.019	0.003	5.56E-12	-0.001	0.001	0.087
rs6550835	G	A	0.006	0.002	9.71E-04	0.025	0.003	2.44E-17	0.000	0.001	0.349
rs6668048	T	C	-0.012	0.002	6.61E-13	-0.021	0.003	4.24E-15	-0.003	0.001	0.003
rs66954617	A	G	-0.006	0.002	0.002	-0.021	0.003	1.72E-13	0.000	0.001	0.407
rs67482514	G	C	0.008	0.002	4.01E-05	0.018	0.003	3.21E-08	-0.002	0.001	0.025
rs6819372	G	A	0.011	0.002	4.34E-11	0.020	0.003	4.02E-13	0.003	0.001	0.001
rs6860963	C	T	-0.004	0.002	0.058	-0.020	0.003	5.57E-09	0.002	0.001	0.049
rs6903716	G	A	-0.006	0.002	6.77E-04	-0.018	0.003	2.39E-09	-0.001	0.001	0.174
rs702222	C	T	0.006	0.002	6.82E-04	0.020	0.003	5.02E-12	0.001	0.001	0.151
rs7029201	G	A	-0.024	0.002	1.98E-44	-0.028	0.003	9.45E-23	-0.002	0.001	0.008
rs7069887	A	C	-0.001	0.002	0.605	0.023	0.004	7.44E-09	0.001	0.001	0.291

rs7116046	T	C	0.007	0.002	1.16E-04	0.016	0.003	3.27E-08	0.001	0.001	0.092
rs7146434	G	A	0.011	0.002	2.37E-10	0.005	0.003	0.053	-0.004	0.001	3.76E-05
rs7172979	T	G	0.017	0.005	0.005	0.061	0.009	2.47E-11	-0.001	0.003	0.413
rs7248006	C	T	0.005	0.002	0.003	0.019	0.003	1.05E-11	0.000	0.001	0.365
rs72768642	T	C	-0.006	0.003	0.095	-0.031	0.005	1.46E-08	-0.005	0.002	0.006
rs73068339	C	G	0.001	0.002	0.432	0.019	0.003	5.96E-10	0.000	0.001	0.353
rs7312919	C	G	-0.001	0.002	0.726	0.018	0.003	4.83E-10	-0.002	0.001	0.007
rs7573001	C	G	-0.002	0.002	0.224	-0.016	0.003	1.32E-08	0.001	0.001	0.201
rs7640196	C	T	0.007	0.002	8.02E-04	0.017	0.003	3.15E-08	0.002	0.001	0.027
rs7652296	G	A	-0.004	0.002	0.0146	-0.017	0.003	3.51E-09	0.001	0.001	0.172
rs766406	G	T	-0.014	0.002	3.17E-16	-0.006	0.003	0.037	-0.002	0.001	0.041
rs7731260	G	A	-0.006	0.002	3.33E-04	-0.015	0.003	2.50E-08	0.000	0.001	0.475
rs7757476	G	A	-0.019	0.002	1.18E-16	-0.006	0.004	0.1	-0.001	0.001	0.154
rs7941785	G	A	-0.006	0.002	7.28E-04	-0.016	0.003	4.75E-08	-0.001	0.001	0.084
rs7964899	G	A	-0.013	0.002	1.33E-14	-0.010	0.003	1.64E-04	0.001	0.001	0.187
rs799444	T	C	0.006	0.002	0.003	0.018	0.003	2.48E-11	0.000	0.001	0.407
rs8006700	T	A	0.012	0.002	1.32E-10	0.018	0.003	4.96E-10	0.001	0.001	0.084
rs80170948	T	G	0.002	0.005	0.656	0.045	0.007	7.69E-10	0.000	0.002	0.464
rs8051038	A	G	0.009	0.002	3.27E-06	0.019	0.003	1.78E-09	0.001	0.001	0.217
rs889169	A	G	0.005	0.002	0.007	0.016	0.003	2.75E-08	0.000	0.001	0.474
rs9384679	T	C	-0.010	0.002	4.88E-08	-0.027	0.003	7.94E-22	0.000	0.001	0.356

rs9503599	C	T	0.011	0.002	4.90E-10	0.017	0.003	8.05E-10	0.000	0.001	0.451
rs9516855	A	G	0.012	0.004	0.002	0.033	0.006	4.19E-08	0.008	0.002	1.08E-04
rs9527702	A	G	0.023	0.002	7.62E-35	0.013	0.003	3.49E-05	0.003	0.001	0.003
rs9616906	G	A	-0.015	0.002	2.92E-18	-0.008	0.003	0.003	-0.002	0.001	0.037
rs967569	T	C	-0.006	0.002	0.002	-0.018	0.003	8.21E-10	-0.001	0.001	0.134
rs9739070	A	G	0.022	0.002	8.95E-26	0.016	0.003	2.78E-06	0.002	0.001	0.052
rs9888986	G	A	0.008	0.003	0.005	0.024	0.004	3.52E-08	0.002	0.002	0.059

Supplementary Table 18: Selective attrition for educational attainment and intelligence based on sex

	Educational attainment			Intelligence			Comparison between sexes across groups	
	Female available (*n*=2,505)	Male available (*n*=1,283)	p[1]	Female available (*n*=2,045)	Male available (*n*=1,134)	p[2]	p[3]	p[4]
**Ethnicity**								
White	2,107 (95.9)	1,129 (96.7)	0.86	1,831 (97.1)	1,033 (96.5)	0.07	0.47	0.88
Non-white	88 (4.1)	39 (3.3)	0.36	67 (2.9)	31 (3.5)	0.45	0.47	0.63
**Mother education**								
O level or less	407 (18.3)	247 (21.0)	0.06	386 (20.1)	241 (22.6)	0.13	0.15	0.40
A level	602 (27.1)	361 (30.7)	0.03	568 (29.6)	332 (31.1)	0.42	0.08	0.89
Degree	1,215 (54.6)	567 (48.3)	<0.001	965 (50.3)	495 (46.3)	0.04	<0.001	0.39
**Partner education**								
O level or less	537 (24.6)	338 (29.2)	<0.001	483 (25.6)	313 (29.8)	0.02	0.53	0.80
A level	606 (27.9)	343 (29.6)	0.29	568 (30.0)	317 (30.1)	0.99	0.12	0.82
Degree	1,036 (47.5)	478 (41.2)	<0.001	839 (44.4)	422 (40.1)	0.03	0.05	0.62
**Mother occupational status**								
Professional	90 (4.6)	62 (5.8)	0.16	85 (5.0)	60 (6.2)	0.24	0.61	0.83
Managerial/technical	662 (33.8)	384 (36.1)	0.22	605 (35.7)	356 (36.5)	0.69	0.26	0.88
Skilled non-manual	816 (41.7)	427 (40.1)	0.43	689 (40.6)	403 (41.3)	0.74	0.53	0.61

Skilled manual	60 (3.1)	40 (3.8)	0.36	55 (3.2)	37 (3.8)	0.52	0.83	0.99
Partly skilled	277 (14.1)	130 (12.2)	0.15	223 (13.1)	105 (10.8)	0.08	0.40	0.34
Unskilled	53 (2.7)	21 (2.0)	0.26	40 (2.4)	14 (1.4)	0.12	0.57	0.45
**Marital status**								
Single	309 (13.6)	125 (10.5)	<0.001	239 (12.3)	107 (9.9)	0.05	0.22	0.70
First	1,727 (76.3)	918 (77.2)	0.57	1,508 (77.9)	845 (78.5)	0.73	0.25	0.51
Marriage 2 or 3	147 (6.5)	90 (7.6)	0.26	115 (5.9)	80 (7.4)	0.13	0.50	0.96
Widowed/divorced/separated	81 (3.6)	56 (4.7)	0.13	75 (3.9)	45 (4.2)	0.75	0.68	0.61
**Home ownership**								
Mortgage/owned	1,873 (83.6)	1,018 (86.8)	0.02	1,658 (86.4)	940 (88.5)	0.11	0.01	0.24
Privately rented	113 (5.0)	61 (5.2)	0.91	78 (4.1)	52 (4.9)	0.33	0.15	0.82
Council rented	185 (8.3)	68 (5.8)	0.01	132 (6.9)	48 (4.5)	0.01	0.11	0.21
Other	70 (3.1)	26 (2.2)	0.16	51 (2.6)	22 (2.1)	0.39	0.42	0.93
**Car ownership**								
Yes	2,037 (95.3)	1,090 (96.0)	0.36	1,795 (95.8)	999 (96.6)	0.35	0.54	0.55
No	101 (4.7)	45 (4.0)	0.36	78 (4.2)	35 (3.4)	0.35	0.54	0.55
**Mother depressed**								
Yes	149 (7.1)	78 (6.9)	0.88	105 (5.8)	72 (7.0)	0.24	0.12	0.98
No	1,948 (92.9)	1,054 (93.1)	0.88	1,699 (94.2)	954 (93.0)	0.24	0.12	0.98
**Smoked during pregnancy**								
Yes	369 (16.3)	187 (15.6)	0.64	271 (14.0)	139 (12.9)	0.41	0.04	0.07

No	1,892 (83.7)	1,008 (84.4)	0.64	1,664 (86.0)	941 (87.1)	0.41	0.04	0.07
**Parity**								
0	1,039 (46.6)	592 (50.3)	0.05	918 (48.2)	559 (52.4)	0.03	0.32	0.33
1	804 (36.1)	397 (33.7)	0.18	674 (35.4)	347 (32.5)	0.12	0.68	0.59
2+	387 (17.4)	189 (16.0)	0.36	313 (16.4)	161 (15.1)	0.36	0.45	0.57

Note:

[1] Comparison of proportion of males and females with measured variable and educational attainment data.

[2] Comparison of proportion of males and females with measured variable and intelligence data.

[3] Comparison of proportion of females with measured variable across two groups.

4 Comparison of proportion of males with measured variable across two groups.

Supplementary Table 19: Variables included in multiple imputation

Variable	Measure	Age at assessment	Question answered by	Number of items	Sample item	Item response options	Item scoring
Ethnicity of child	1 item	32 weeks gestation	Mother	2	"How would you describe the race or ethnic group of yourself/your partner?"	8 response options, including 'Other (please describe)'.	Child's ethnic background defined as non-white if either mother or father are non-white
Mother's age at first pregnancy	1 item	18 weeks gestation	Mother	1	"How old were you when you became pregnant for the very first time?"	Open response.	Higher score signifies higher age
Homeownership status	1 item	8 months	Mother	1	"Do you currently live in.."	6 response options including "Mortgaged" and "Rented from private landlord"	Higher score signifies greater home ownership
Mother marital status	1 item	32 weeks gestation	Mother	1	"What is your present marital status?	6 response options including "Married" and "Separated"	High score signifies currently married
Mother and partner educational qualifications	2 items	32 weeks gestation	Mother	2	"What educational qualifications do you/your partner have?"	List of qualifications, respondent must tick all that apply	Higher score signifies more educational qualifications
Maternal smoking during pregnancy	1 item	32 weeks gestation	Mother	1	"How many cigarettes per day are you yourself smoking?"	Open response	Greater tar intake
Maternal depression	Edinburgh Postnatal Depression Scale[a]	32 weeks gestation	Mother	10	"Felt sad/miserable in past week"	4-point scale ranging, including 'Yes, most of the time"	Greater depressive symptoms

Adolescent depressive symptoms	Moods and Feelings Questionnaire[b]	13	Child	9	"Teenager felt miserable or unhappy"	3-pont scale ranging from "Not at all" to "True"	Greater depressive symptoms
Childhood IQ	Wechsler Intelligence Scale for Children (WISC)[c]	8	Child	-	-	Responses range from 45 to 151.	Higher IQ

Note: Multiple imputation was conducted using these sociodemographic factors. These were selected as they have previously been associated with missingness in ALSPAC[d]. In total, 60 imputations were ran using Chained Equations (MICE).

[a] Cox, J. L., Holden, J. M., & Sagovsky, R. (1987). Detection of postnatal depression: Development of the 10-item Edinburgh Postnatal Depression Scale. *British Journal of Psychiatry, 150,* 782-786.

[b] Angold, A., Costello, E. J., Messer, S. C., Pickles, A., Winder, F., & Silver, D. (1995). The development of a short questionnaire for use in epidemiological studies of depression in children and adolescents. *International Journal of Methods in Psychiatric Research*, 5, 237-249.

[c] Wechsler, D. (1949). Wechsler Intelligence Scale for Children. San Antonio, TX, US: Psychological Corporation

[d] Houtepen, L. C., Heron, J., Suderman, M. J., Tilling, K., & Howe, L. D. (2018). Adverse childhood experiences in the children of the Avon Longitudinal Study of Parents and Children (ALSPAC). Wellcome open research, 3, 106. https://doi.org/10.12688/wellcomeopenres.14716.1.

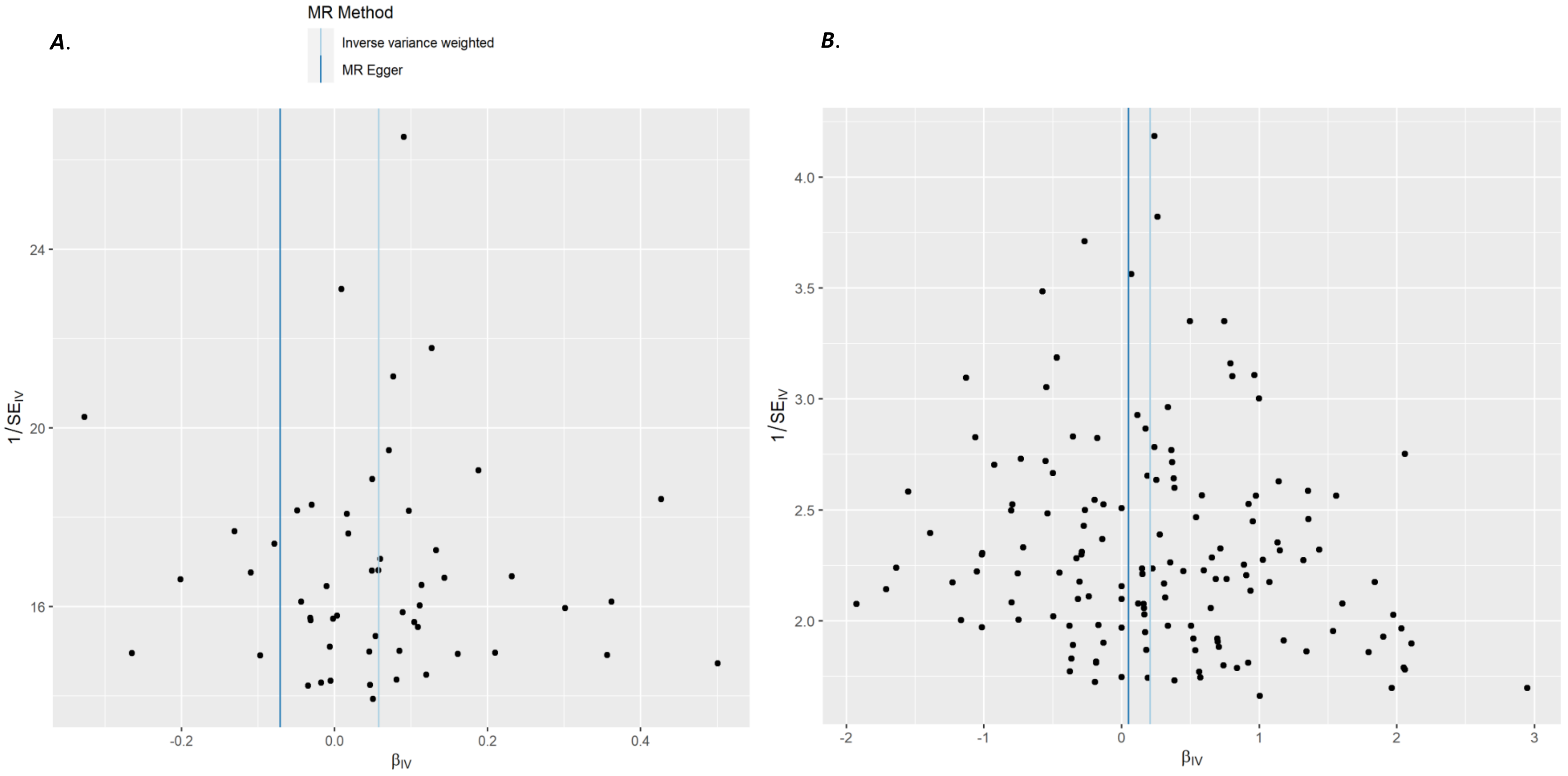

Supplementary Figure 1: Funnel plot assessing the extent to which pleiotropy is balanced across the set of instruments used in the univariate MR analysis of A) years of schooling on wellbeing and B) wellbeing on years of schooling. βIV represents the effect size of each SNP, and 1/SEIV represents the inverse standard error for each SNP effect.

***A*.**

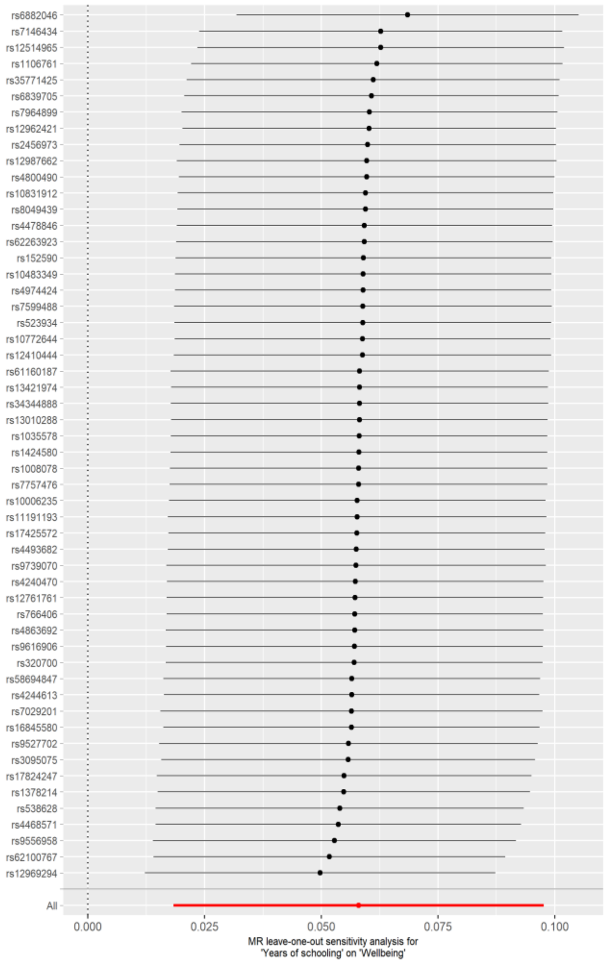

***B*.**

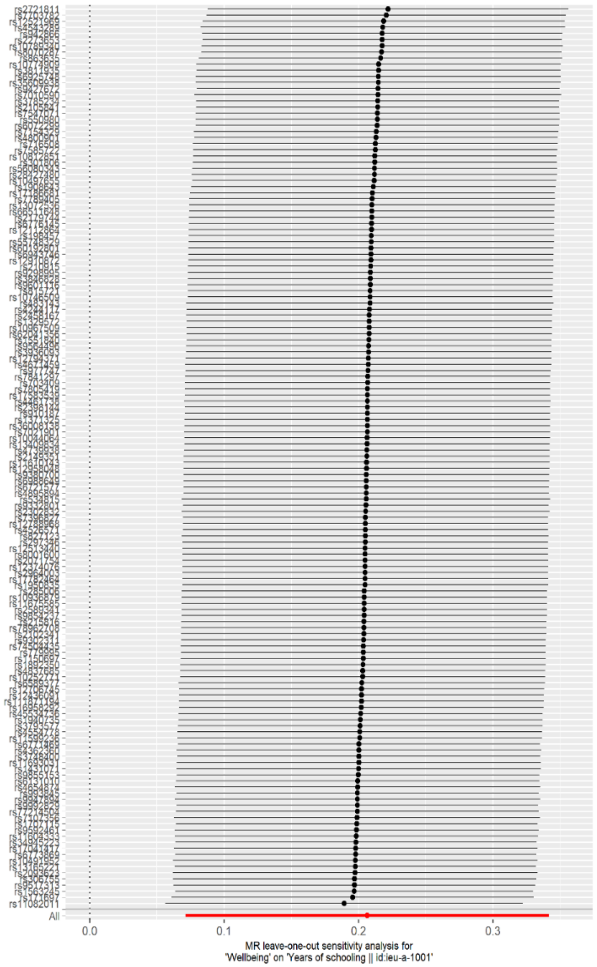

Supplementary Figure 2: Leave-one-out analyses for A) two-sample univariable MR analysis of years of schooling on wellbeing and B) two-sample univariable MR analysis of wellbeing on years of schooling. As evident in both forest plots, neither of the univariable associations were driven by a single SNP.

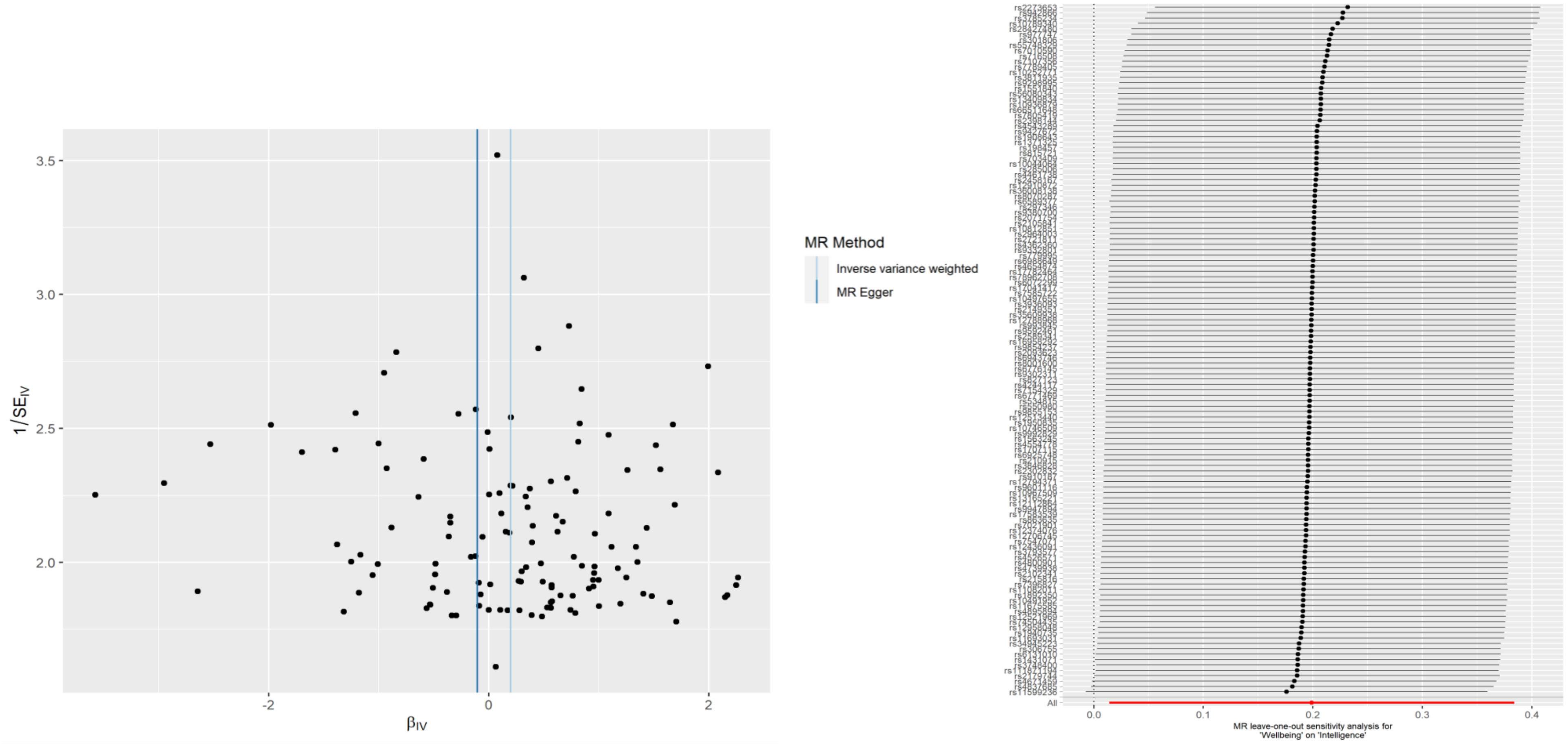

Supplementary Figure 3: A) Funnel plot assessing the extent to which pleiotropy is balanced across the set of instruments used in the univariate MR analysis of wellbeing on intelligence. βIV represents the effect size of each SNP, and 1/SEIV represents the inverse standard error for each SNP effect. B) Leave-one-out analyses for two-sample univariable MR analysis of wellbeing on intelligence. As evident in both the funnel and forest plots, the univariable association did not appear to be not driven by a single SNP.

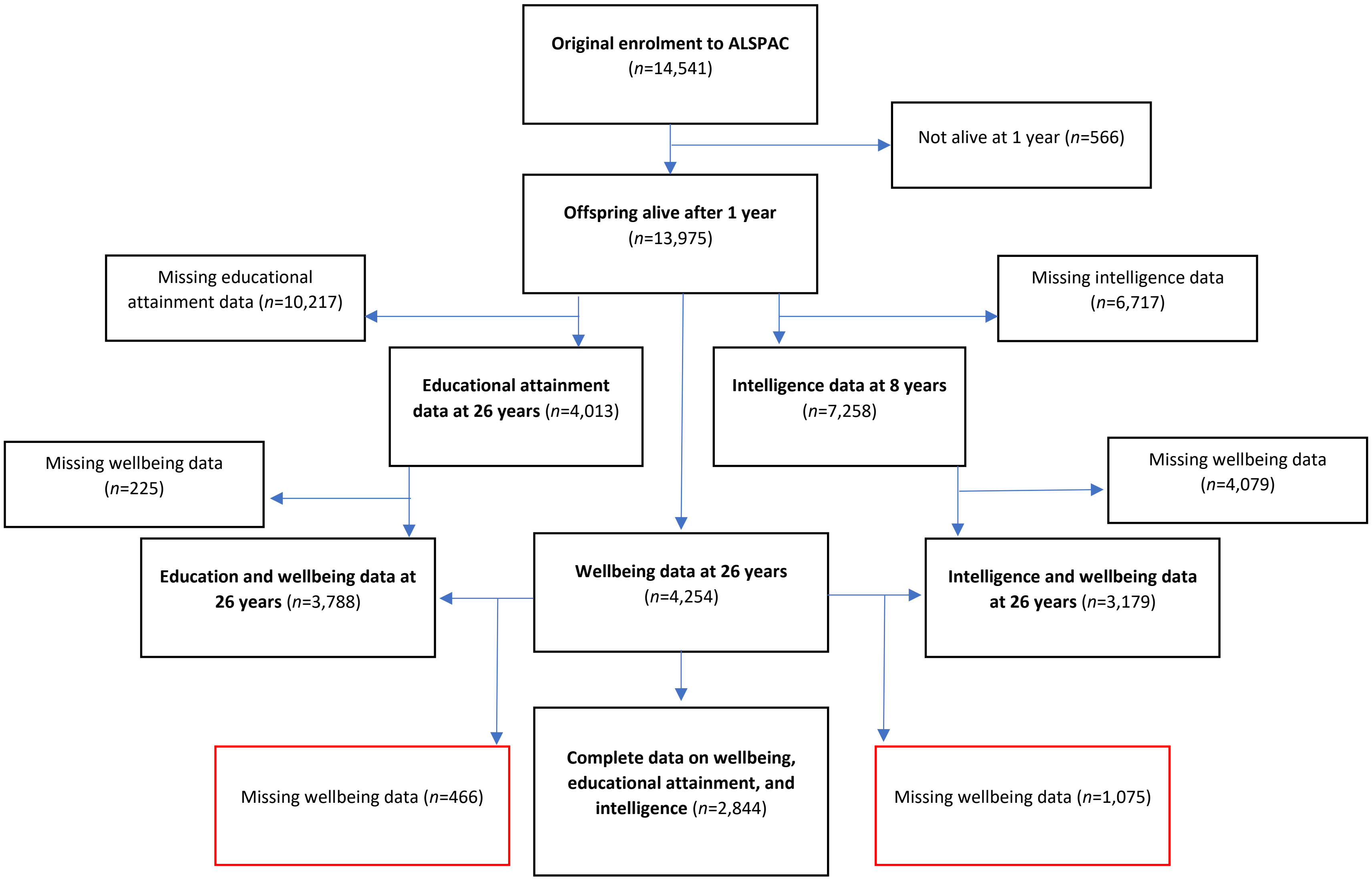

Supplementary Figure 4: Flowchart of data available for use in ALSPAC. Boxes in red represent data imputed during multiple imputation.
